# Cannabinoid CB1 Receptor Regulates Spinal Dorsal Horn Synaptic Plasticity in a Mouse Model of Neuropathic Pain through the CB1R/SREBP1/NAMPT/SIRT1 Metabolic Regulatory Framework

**DOI:** 10.1002/brb3.71837

**Published:** 2026-10-13

**Authors:** Lu Liu, Chenglong Wu, Hui Xia, Jie Cheng, Liang Zhong, Jiang Wu

**Affiliations:** ^1^ Department of Anesthesiology Wuhan Children's Hospital Wuhan China; ^2^ Department of Pain Wuhan Fourth Hospital Wuhan China

**Keywords:** CB1R, NAMPT, NP, SIRT1, SREBP1, synaptic plasticity

## Abstract

**Background:**

Neuropathic pain is sustained by maladaptive synaptic plasticity in the spinal dorsal horn. Increasing evidence suggests that metabolic regulation is closely linked to synaptic function, as lipid metabolism, membrane microdomain organization, NAD^+^‐dependent signaling, and cellular stress‐response pathways can influence receptor trafficking, neuronal excitability, and activity‐dependent synaptic remodeling. Because cannabinoid CB1 receptor (CB1R) signaling participates in both pain processing and metabolic control, we investigated whether CB1R modulation is associated with coordinated changes in spinal synaptic plasticity and metabolic regulators.

**Methods:**

A chronic constriction injury (CCI) mouse model was used. Mice were treated with the CB1R antagonist AM251 or the CB1R agonist WIN55,212‐2. Mechanical and thermal hypersensitivity, C‐fiber–evoked long‐term potentiation in the spinal dorsal horn, plasma lipid indices, and the expression of CB1R, SREBP1, NAMPT, and SIRT1 were assessed.

**Results:**

CB1R activation alleviated CCI‐induced mechanical and thermal hypersensitivity and attenuated exaggerated C‐fiber–evoked spinal long‐term potentiation, whereas CB1R inhibition produced the opposite tendency. AM251 reduced spinal dorsal horn CB1R, SREBP1, and NAMPT protein levels by 49.0%, 37.8%, and 57.5%, respectively, compared with untreated CCI mice, while increasing SIRT1 protein by 17.8% (*p* < 0.01). In contrast, WIN55,212‐2 increased CB1R, SREBP1, and NAMPT protein levels by 24.5%, 23.7%, and 17.2%, respectively, and reduced SIRT1 protein by 28.8% compared with untreated CCI mice (*p* < 0.01). qPCR analysis showed consistent changes at the transcriptional level.

**Conclusion:**

CB1R activation is associated with pain relief, suppression of spinal synaptic potentiation, and coordinated regulation of the SREBP1/NAMPT/SIRT1 metabolic axis. These findings suggest that CB1R‐mediated metabolic regulation may contribute to maladaptive spinal synaptic plasticity in neuropathic pain.

## Graphical abstract

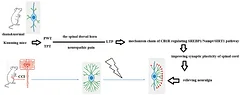

## 1 Introduction

Neuropathic pain (NP) is a chronic and debilitating condition resulting from injury or dysfunction of the nervous system (Cavalli et al. 2019; Colloca et al. 2017). A central pathological feature of NP is central sensitization, which is characterized by enhanced excitability and synaptic plasticity within the spinal dorsal horn (SDH) (Latremoliere and Woolf 2009). In particular, long‐term potentiation (LTP) of nociceptive synaptic transmission in superficial dorsal horn laminae is widely regarded as a cellular substrate underlying persistent pain hypersensitivity and the maintenance of chronic pain states (Latremoliere and Woolf 2009; Chen and Tang 2024; Kurabe et al. 2022).

The endogenous cannabinoid system plays an important role in modulating pain processing at both peripheral and central levels (Anthony et al. 2020; Campos et al. 2021). Among cannabinoid receptors, the cannabinoid Type 1 receptor (CB1R) is abundantly expressed in the central nervous system and has been implicated in the regulation of synaptic transmission, neuronal excitability, and pain‐related behaviors (Milligan et al. 2020; Zou and Kumar 2018). Pharmacological activation of CB1R has been shown to alleviate neuropathic pain in various experimental models, whereas CB1R inhibition can exacerbate pain hypersensitivity (Anthony et al. 2020; Campos et al. 2021). Despite extensive evidence supporting the involvement of CB1R in pain modulation, the downstream mechanisms linking CB1R signaling to maladaptive synaptic plasticity in the SDH remain incompletely understood. It should be noted that CB1R exerts complex and context‐dependent effects in the nervous system, depending on cell type, anatomical location, ligand exposure, and disease state; therefore, the present study focused on the overall modulatory effect of pharmacological CB1R intervention in the SDH rather than on a single cell‐type‐specific mechanism.

Beyond their role as energy substrates, lipids are essential structural components of neuronal membranes and lipid rafts—cholesterol‐ and sphingolipid‐enriched microdomains that organize membrane receptors and intracellular signaling complexes (Grassi et al. 2020; Tsui‐Pierchala et al. 2002). Lipid rafts play a critical role in receptor localization, trafficking, and signal transduction, thereby shaping synaptic transmission and synaptic plasticity (Tsui‐Pierchala et al. 2002). Alterations in membrane lipid composition can modify the local microenvironment surrounding synaptic receptors and ion channels, ultimately influencing neuronal excitability and synaptic strength (Grassi et al. 2020). Increasing evidence suggests that dysregulated lipid metabolism is associated with altered neuronal function, neurodegenerative processes, and changes in pain sensitivity. Because CB1R is a key regulator of lipid metabolism at both systemic and cellular levels, CB1R‐mediated metabolic alterations may indirectly affect synaptic plasticity by reshaping membrane lipid environments in pain‐processing circuits (Bari et al. 2005; Saumell‐Esnaola et al. 2021).

Alterations in membrane lipid composition (e.g., cholesterol and sphingolipids) modify lipid raft formation, which is essential for NMDA receptor clustering and downstream Ca^2^^+^ signaling—key events in LTP induction. SREBP1, a master regulator of lipid synthesis, directly controls the production of these membrane lipids (Chen et al. 2017). Nicotinamide phosphoribosyltransferase (NAMPT), the rate‐limiting enzyme in nicotinamide adenine dinucleotide (NAD^+^) biosynthesis, links cellular metabolic state to redox balance and stress responses (Kim et al. 2014; Zhang et al. 2025). Sirtuin 1 (SIRT1), an NAD^+^‐dependent deacetylase, functions as a metabolic sensor and has been reported to influence synaptic function and neuronal plasticity under both physiological and pathological conditions (Michan et al. 2010). Although previous studies have examined spinal LTP or metabolic alterations in NP, few have systematically integrated electrophysiological evidence with metabolic signaling to clarify how CB1R‐associated metabolic regulation may contribute to maladaptive synaptic plasticity in the SDH under neuropathic conditions (Martinez Ramirez et al. 2023).

Based on these considerations, we hypothesized that CB1R modulation is associated with coordinated metabolic alterations, reflected by changes in systemic lipid indices and the expression of SREBP1, NAMPT, and SIRT1 in the SDH, which may contribute to synaptic plasticity changes in neuropathic pain. Rather than defining a strictly linear signaling cascade, this study considered SREBP1, NAMPT, and SIRT1 as components of a CB1R‐associated functional framework. We therefore aimed to determine whether these metabolic regulators undergo coordinated changes following CB1R modulation and whether such changes are associated with altered spinal synaptic plasticity. Defining this CB1R‐associated metabolic framework may provide mechanistic insight into neuropathic pain maintenance and help identify metabolism‐related targets for therapeutic intervention. To test this hypothesis, we employed a chronic constriction injury (CCI) model in mice and combined behavioral assessments, spinal electrophysiological recordings, metabolic profiling, and molecular analyses.

## 2 Materials and Methods

### 2.1 Animals

A total of 68 male KM mice (6 weeks old, 20–25 g) were used. Only male mice were included to minimize estrous cycle‐related variability in pain sensitivity and endogenous cannabinoid signaling. All animal procedures were approved by the Wuhan University Animal Laboratory Center (AUP2019073) and performed in accordance with AAALAC and IACUC guidelines. Detailed housing conditions are provided in the Supplementary Methods.

### 2.2 CCI Model and Drug Administration

A CCI model of neuropathic pain was established by ligating the left sciatic nerve under sodium pentobarbital anesthesia: three loose chromic catgut ligatures were placed with 1 mm spacing, and stable mechanical/thermal hypersensitivity at Day 7 confirmed successful modeling. Sham mice underwent identical surgery without nerve ligation.

Mice were assigned to 9 groups (normal control, sham, CCI, each with vehicle, AM251 or WIN55,212‐2 treatment). AM251 (5 mg/kg, twice daily) (Ueda et al. 2014; Ostadhadi et al. 2016; O'Keefe et al. 2022) or WIN55,212‐2 (1 mg/kg, three times daily) (Chesworth et al. 2026; Cajiao‐Manrique et al. 2023)was administered intraperitoneally from day 11 to 13 post‐surgery. Detailed surgical procedures and group allocation are described in the Supplementary Methods.

### 2.3 Behavioral Assessments

Mechanical and thermal hypersensitivity were assessed at baseline, Day 7, and Day 14 post‐surgery. Mechanical hypersensitivity was evaluated by paw withdrawal frequency using von Frey filaments, a graded approach suitable for detecting response probability changes in mouse neuropathic pain models. Thermal hypersensitivity was measured as paw withdrawal latency with a plantar test apparatus. Detailed testing parameters are provided in the Supplementary Methods.

### 2.4 In Vivo Electrophysiological Recordings

In vivo extracellular field potentials evoked by A‐ and C‐fiber stimulation were recorded in laminae I–II of the ipsilateral SDH (T13–L1) under anesthesia. A‐ and C‐fiber components were distinguished by conduction latency (2–5 ms vs. 18–35 ms). LTP was induced by high‐frequency stimulation (HFS) as previously described (Liu and Sandkühler 1995; Sandkühler and Liu 1998), and quantified by changes in peak amplitude and response area. After applying predefined quality exclusion criteria, 64 mice were included in the final analysis. Detailed recording parameters are provided in the Supplementary Methods.

### 2.5 Quantitative Assay Kit Measurements

Plasma levels of TG, TC, LDL‐C, and HDL‐C were measured using commercial colorimetric assay kits (Abcam, Cambridge, UK). Detailed blood collection procedures and assay protocols are provided in the Supplementary Methods.

### 2.6 Molecular Analyses

Ipsilateral lumbar SDH (L3–L5) was harvested for molecular detection. Protein expression of CB1R, SREBP1, NAMPT, and SIRT1 was assessed by immunohistochemistry (IHC, with PBS‐incubated negative controls) and Western blotting, with GAPDH as the loading control. Uncropped Western blot images are provided in the Supplementary Materials.

mRNA levels were quantified by RT‐qPCR with β‐actin as the internal reference, using a SYBR green‐based system. Detailed protocols, antibody specifications, and primer sequences are listed in the Supplementary Methods.

### 2.7 Statistical Analysis

Sample sizes were calculated as previously described (Charan and Kantharia 2013). Statistical analysis was performed using SPSS 23.0. Data are presented as mean ± standard deviation (SD). Longitudinal behavioral data were analyzed by two‐way repeated‐measures ANOVA followed by Tukey's post hoc test. Other multi‐group comparisons were performed using one‐way ANOVA with Tukey–Kramer post hoc tests. *P* < 0.05 was considered statistically significant.

## 3 Results

### 3.1 CB1R Modulation Alters Mechanical and Thermal Hypersensitivity After CCI

To determine whether CB1R modulation affects pain‐related behaviors after nerve injury, mechanical and thermal hypersensitivity were assessed longitudinally after CCI. Mice were treated with the CB1R antagonist AM251 or the CB1R agonist WIN55,212‐2.

Baseline values did not differ among groups for either endpoint (Tables S1–S2). For thermal hypersensitivity, AM251 treatment exacerbated the CCI‐induced reduction in paw withdrawal latency (TWL), which decreased to 4.25 ± 1.0 s at day 14 (66.5% reduction from baseline). At Day 14, TWL was significantly lower in the CCI + AM251 group than in the sham + AM251 and normal + AM251 groups (*p* < 0.01). WIN55,212‐2 partially reversed this effect, increasing TWL to 9.1 ± 1.6 s (114% increase vs. CCI + AM251, *p* < 0.01) (Table S2, Figure 1A).

**FIGURE 1:**
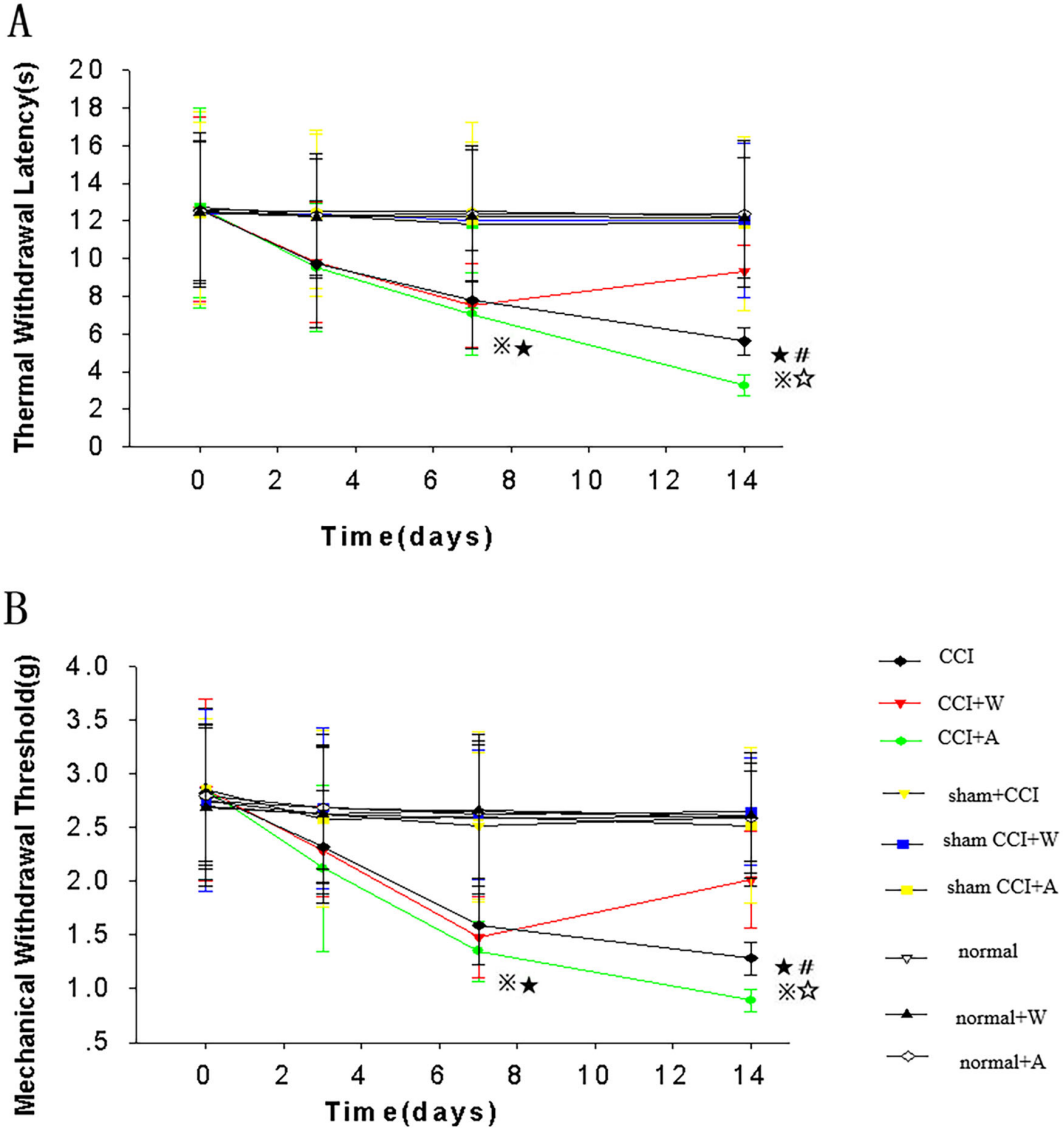
CB1R modulation alters mechanical and thermal hypersensitivity in mice with chronic constriction injury (CCI). (A) Time course of thermal pain threshold (TPT) assessing thermal hypersensitivity. (B) Time course of paw withdrawal threshold (PWT) assessing mechanical hypersensitivity. Mice were treated with vehicle, AM251 (CB1R antagonist), or WIN55, 212‐2 (CB1R agonist) from Day 11 to 13 post‐surgery. Data are presented as mean ± SD. ^*^ *P* < 0.05, CCI group vs the sham group(+A, +W), normal group(+A, +W), ^★^ *P* < 0.01, CCI+A group vs the sham+A(CCI, +W) and normal+A(CCI, +W) groups. ^※^ *P* < 0.01, CCI+W group vs sham+W(CCI, +A) and normal+W(CCI, +A) groups. ^☆^ *P* < 0.01 CCI+A group vs CCI+W group. ^#^ *P* < 0.05, CCI group vs CCI+A or CCI+W group. (*n* = 6–9 per group).

A similar pattern was observed for mechanical paw withdrawal threshold (PWT). AM251 aggravated CCI‐induced mechanical hypersensitivity, with PWT dropping to 0.89 ± 0.21 g at Day 14. WIN55,212‐2 treatment increased PWT to 2.01 ± 0.45 g, representing a 126% improvement compared with the CCI + AM251 group (*p* < 0.01). Full time‐course data are provided in Table S1 and Figure 1B.

Collectively, these results indicate that CB1R antagonism aggravates CCI‐induced pain hypersensitivity, whereas CB1R activation partially reverses these behavioral changes.

### 3.2 CB1R Modulation Alters A‐Fiber– and C‐Fiber–Evoked Spinal LTP

To assess spinal synaptic plasticity after nerve injury, A‐fiber– and C‐fiber–evoked field potentials were recorded from the SDH on Day 14 after CCI. Representative traces and time‐course plots are shown in Figure 2A–C. LTP was quantified using both peak amplitude and response area before and 60 min after HFS.

**FIGURE 2:**
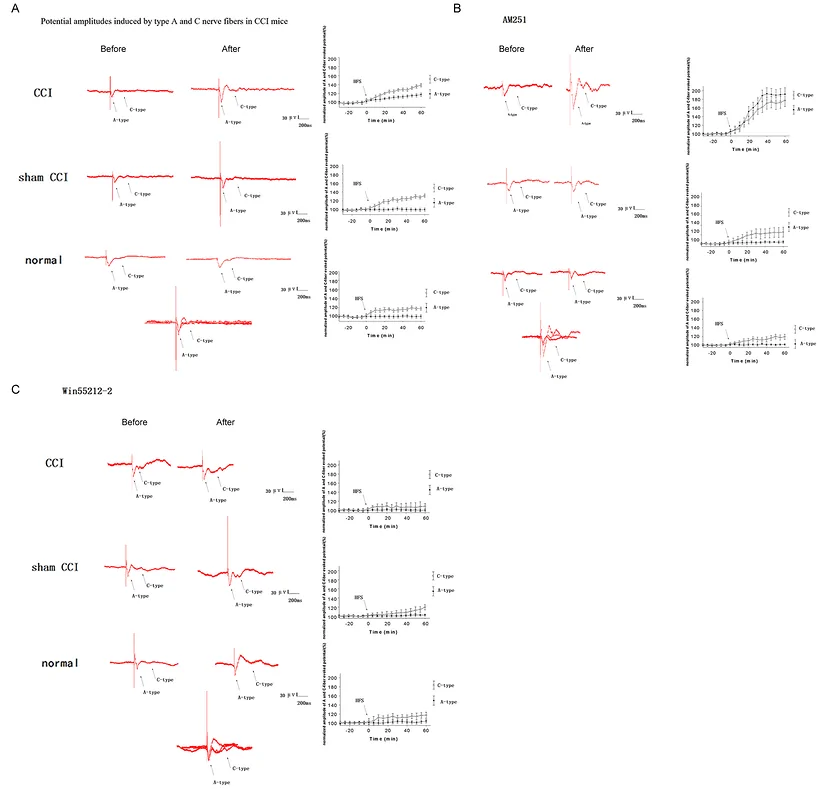
CB1R activation attenuates C‐fiber–evoked synaptic potentiation in the spinal dorsal horn. (A) Representative field‐potential traces and time‐course quantification of A‐fiber– and C‐fiber–evoked responses in untreated groups, including CCI, sham CCI, and normal control mice. (B) Representative field‐potential traces and time‐course quantification of A‐fiber– and C‐fiber–evoked responses in AM251‐treated groups, including CCI + AM251, sham CCI + AM251, and normal control + AM251 mice. (C) Representative field‐potential traces and time‐course quantification of A‐fiber– and C‐fiber–evoked responses in WIN55,212‐2–treated groups, including CCI + WIN55,212‐2, sham CCI + WIN55,212‐2, and normal control + WIN55,212‐2 mice. For each group, representative traces before and after high‐frequency stimulation (HFS) are shown. HFS was delivered at time 0. A‐fiber– and C‐fiber–evoked responses were distinguished according to conduction latency, with A‐fiber responses defined as responses occurring within 2–5 ms after stimulation and C‐fiber responses defined as responses occurring within 18–35 ms after stimulation. LTP was quantified as the percentage change in evoked response amplitude and/or response area relative to the 30‐min pre‐HFS baseline. Representative traces show examples from a single recording session, whereas the time‐course plots and quantitative analyses were generated from one baseline period and one post‐HFS recording period per animal. A‐ and C‐fiber components were extracted from the same evoked field potential according to conduction latency. Data are presented as mean ± SD. *n* = 6 for CCI, *n* = 5 for sham CCI, *n* = 5 for normal control, *n* = 8 for CCI + AM251, *n* = 7 for sham CCI + AM251, *n* = 8 for normal + AM251, *n* = 9 for CCI + WIN55,212‐2, *n* = 9 for sham CCI + WIN55,212‐2, and *n* = 7 for normal + WIN55,212‐2.

For A‐fiber–evoked responses, HFS markedly increased peak amplitude in CCI + AM251 mice from 17.4 ± 3.8 µV to 59.5 ± 12.3 µV, corresponding to a 240.9% increase (*p* < 0.01; Table S3, Figure 2A–C). In contrast, the CCI + WIN55,212‐2 group showed only a slight, non‐significant increase from 13.7 ± 3.8 µV to 14.5 ± 3.2 µV, corresponding to a 5.8% increase (*p* > 0.05; Table S3). A‐fiber response area showed a similar pattern, increasing from 47.8 ± 8.5 µV·s to 91.6 ± 18.7 µV·s in CCI + AM251 mice, but only from 42.4 ± 8.7 µV·s to 43.2 ± 9.4 µV·s in CCI + WIN55,212‐2 mice (92.6% vs. 1.9%; Table S4).

For C‐fiber–evoked responses, CCI + AM251 mice showed robust potentiation, with peak amplitude increasing from 14.2 ± 3.8 µV to 42.4 ± 7.8 µV, corresponding to a 198.6% increase (*p* < 0.001; Table S5, Figure 2A–C). WIN55,212‐2 reduced this potentiation, with C‐fiber amplitude increasing from 13.7 ± 3.8 µV to 21.5 ± 4.2 µV, corresponding to a 57.1% increase (*p* < 0.05; Table S5). C‐fiber response area increased from 59.5 ± 15.2 µV·s to 105.7 ± 26.1 µV·s in CCI + AM251 mice, but only from 55.4 ± 8.7 µV·s to 60.5 ± 9.9 µV·s in CCI + WIN55,212‐2 mice (77.6% vs. 5.1%; Table S6). WIN55,212‐2 markedly suppressed HFS‐induced potentiation of both A‐ and C‐fiber responses. For peak amplitude, WIN55,212‐2 reduced A‐fiber potentiation by 97.6% and C‐fiber potentiation by 71.2% relative to AM251 treatment (Tables S3, S5). Response area followed a similar inhibitory trend (Tables S4, S6). This effect was not selective for C‐fibers, although the C‐fiber component remains pathophysiologically relevant as it reflects nociceptive afferent input.

### 3.3 Systemic Lipid Indices are Altered Following CB1R Modulation

To determine whether CB1R modulation was accompanied by systemic metabolic changes, plasma TG, TC, LDL, and HDL levels were measured after pharmacological treatment. In AM251‐treated CCI mice, TG, TC, and LDL levels were lower than those in sham + AM251 and normal + AM251 mice, whereas HDL was higher. Specifically, CCI + AM251 mice showed TG of 1.10 ± 0.50 mmol/L, TC of 2.78 ± 0.30 mmol/L, LDL of 1.40 ± 0.50 mmol/L, and HDL of 1.90 ± 0.70 mmol/L (Table S7, Figure 3A–D).

**FIGURE 3:**
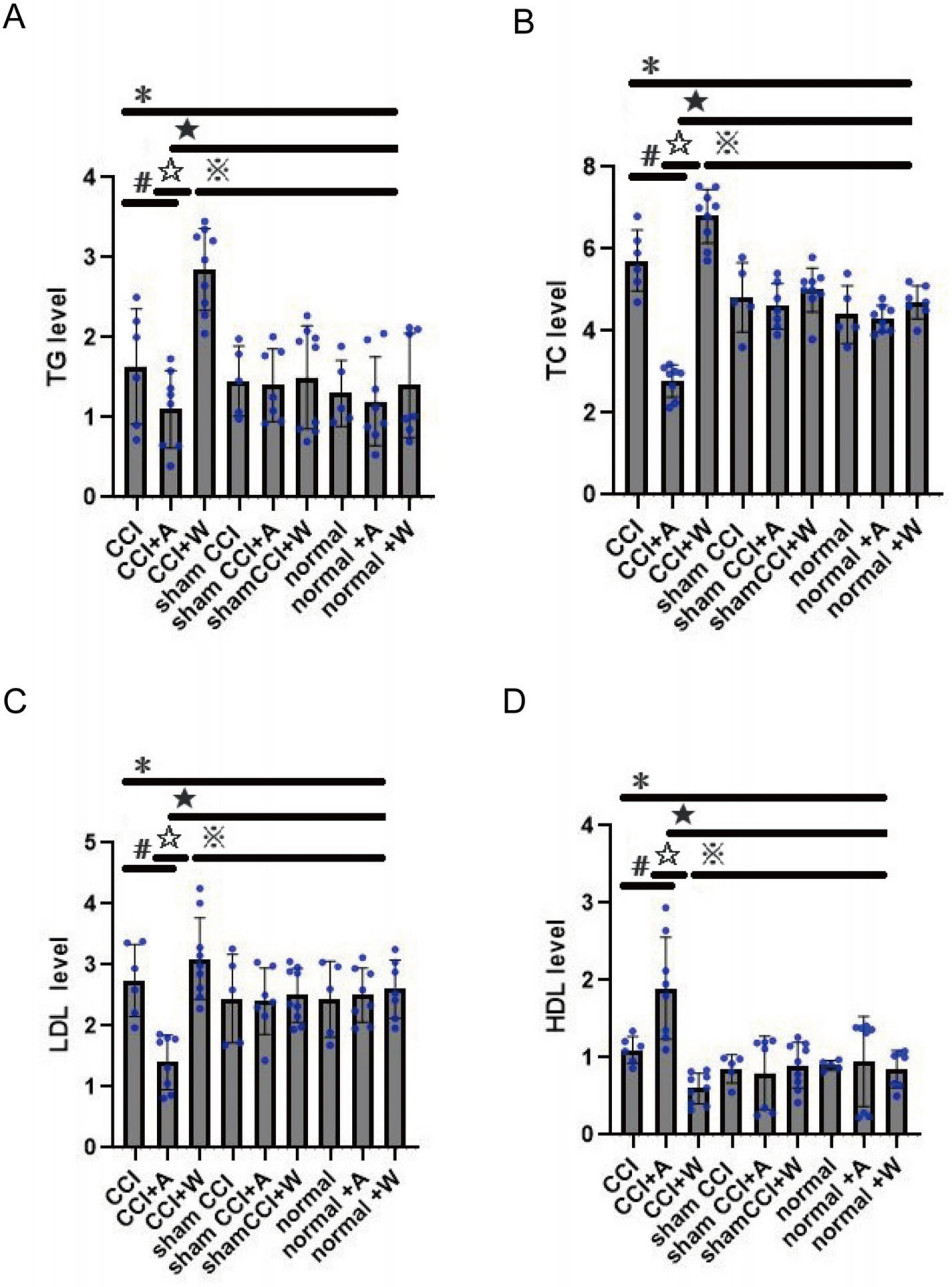
Systemic lipid profiles are altered following CB1R modulation in neuropathic pain mice. Plasma levels of (A) triglycerides (TG), (B) total cholesterol (TC), (C) low‐density lipoprotein (LDL), and (D) high‐density lipoprotein (HDL) were measured using commercial colorimetric assay kits at the end of the experiment (Day 14). CB1R antagonism (AM251) led to decreased TC and LDL but increased TG and HDL levels in CCI mice. Data are presented as mean ± SD. *n* = 8 for CCI + AM251, *n* = 7 for sham CCI + AM251, *n* = 8 for normal + AM251, *n* = 9 for CCI + WIN55,212‐2, *n* = 9 for sham CCI + WIN55,212‐2, and *n* = 7 for normal + WIN55,212‐2. Comparisons among groups were performed using one‐way ANOVA followed by Tukey–Kramer post hoc test. ★*p* < 0.05 or *p* < 0.01 vs the corresponding sham and normal control groups; ※*p* < 0.05 or *p* < 0.01 vs the corresponding sham + WIN55,212‐2 and normal + WIN55,212‐2 groups; ☆*p* < 0.01 vs the CCI + WIN55,212‐2 group.

In contrast, WIN55,212‐2‐treated CCI mice showed higher TG, TC, and LDL levels but lower HDL levels than the corresponding sham and normal groups. In the CCI + WIN55,212‐2 group, TG increased to 2.85 ± 0.50 mmol/L, TC to 6.80 ± 0.50 mmol/L, and LDL to 3.10 ± 0.60 mmol/L, whereas HDL decreased to 0.60 ± 0.20 mmol/L (Table S7, Figure 3A–D). Direct comparison between CCI treatment groups showed that WIN55,212‐2 increased TG, TC, and LDL by 159.1%, 144.6%, and 121.4%, respectively, while reducing HDL by 68.4% compared with AM251 treatment. These findings indicate that CB1R modulation is associated with systemic lipid remodeling after CCI.

### 3.4 CB1R Modulation is Associated with Coordinated Changes in Metabolic Regulator Expression in the Spinal Dorsal Horn

Consistent changes in CB1R, SREBP1, NAMPT and SIRT1 expression were observed across immunohistochemistry, Western blot and qPCR assays (Figures 4, 5, 6, Tables 1, 2, Table S8). At the protein level, AM251 reduced CB1R, SREBP1 and NAMPT by 49.0%, 37.8% and 57.5% respectively, and increased SIRT1 by 17.8% relative to untreated CCI mice (all *p* < 0.01); WIN55,212‐2 produced the opposite effect. Transcriptional changes paralleled the protein findings with consistent statistical significance.

**FIGURE 4:**
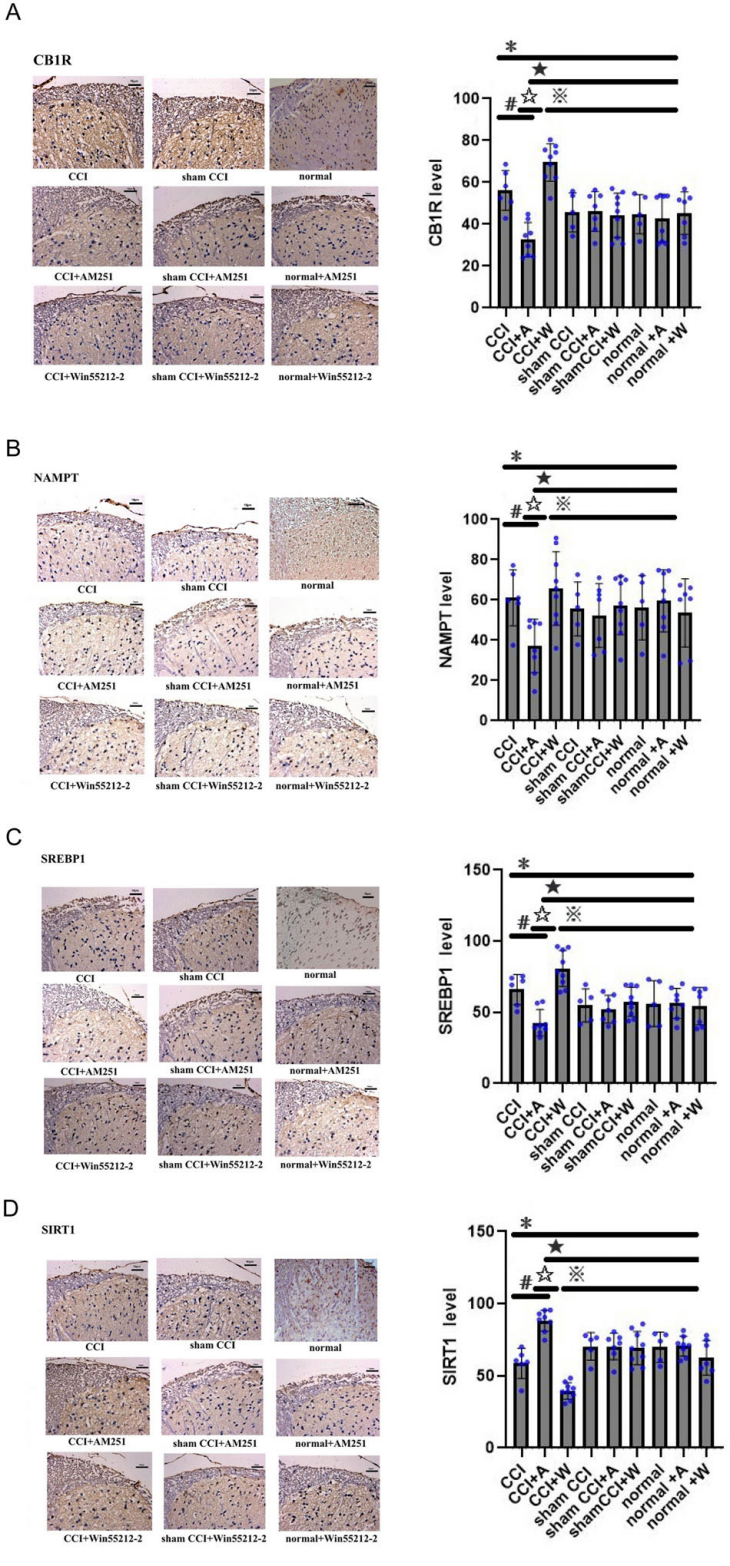
Expression of CB1R, NAMPT, SREBP1, and SIRT1 in the spinal dorsal horn. Representative immunohistochemical images and quantitative analysis of (A) CB1R, (B) NAMPT, (C) SREBP1, and (D) SIRT1 in the ipsilateral lumbar spinal dorsal horn (L3–L5). Scale bar = 50 µm. Images were captured at 200× magnification. The bar graphs represent the mean optical density (MOD) of the immunoreactivity. CB1R inhibition (AM251) decreased CB1R, NAMPT, and SREBP1 but increased SIRT1 expression. Data are presented as mean ± SD. *n* = 8 for CCI + AM251, *n* = 7 for sham CCI + AM251, *n* = 8 for normal + AM251, *n* = 9 for CCI + WIN55, 212‐2, *n* = 9 for sham CCI + WIN55, 212‐2, and *n* = 7 for normal + WIN55, 212‐2. For each animal, three to five fields within laminae I–II of the ipsilateral spinal dorsal horn were analyzed. Group comparisons were performed using one‐way ANOVA followed by Tukey–Kramer post hoc test. ^*^ *P* < 0.05, CCI group vs the sham group(+A, +W), normal group(+A, +W), ^★^ *P* < 0.01, CCI+A group vs the sham+A(CCI, +W) and normal+A(CCI, +W) groups. ^※^ *P* < 0.01, CCI+W group vs sham+W(CCI, +A) and normal+W(CCI, +A) groups. ^☆^ *P* < 0.01 CCI+A group vs CCI+W group. ^#^ *P* < 0.05, CCI group vs CCI+A or CCI+W group.

**FIGURE 5:**
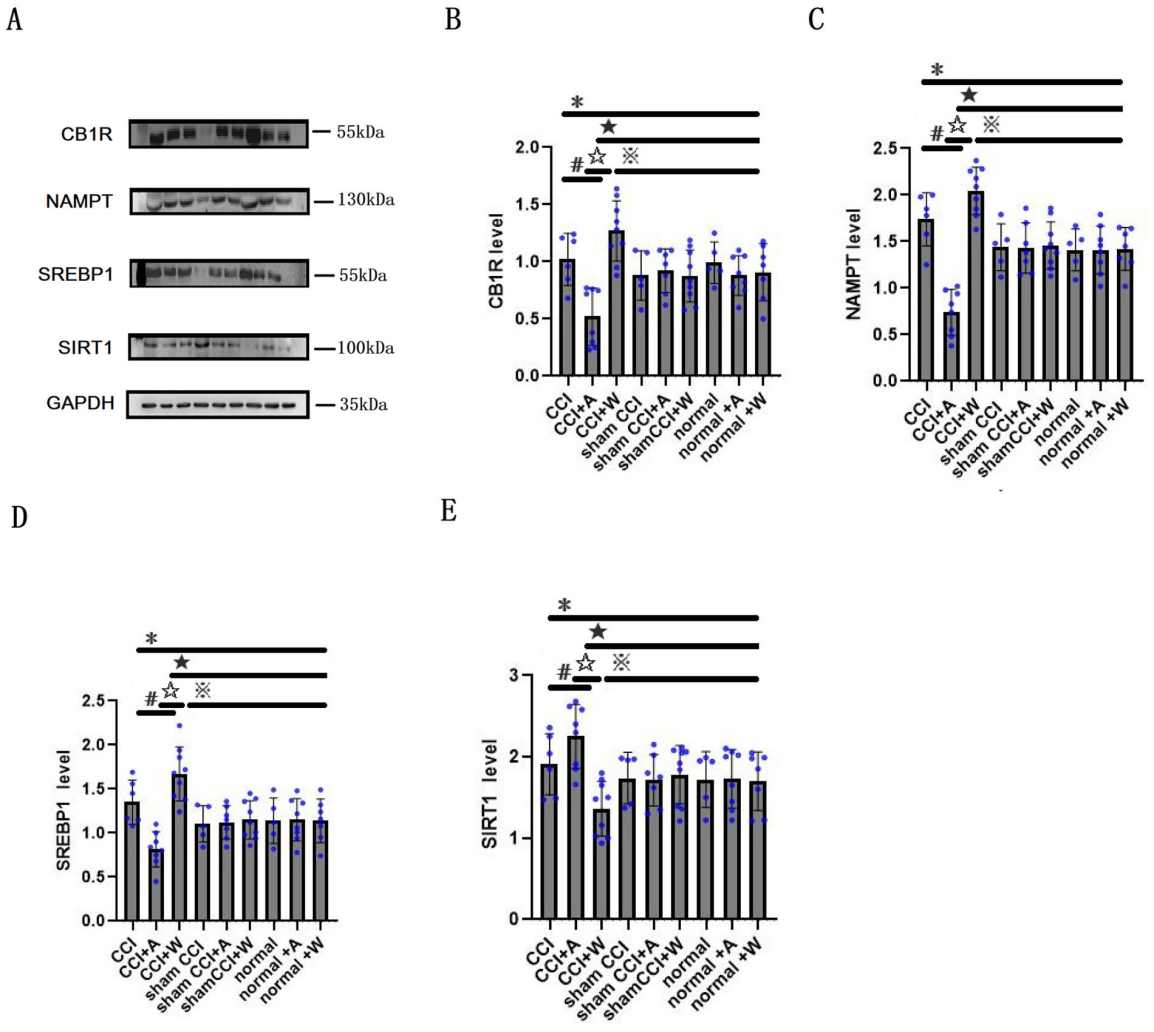
Western blot analysis of protein expression in the spinal dorsal horn. (A) Representative western blot bands showing the expression of CB1R, NAMPT, SREBP1, and SIRT1. GAPDH was used as a loading control. (B–E) Densitometric quantification of the relative protein expression of (B) CB1R, (C) NAMPT, (D) SREBP1, and (E) SIRT1. Data are presented as mean ± SD. *n* = 8 for CCI + AM251, *n* = 7 for sham CCI + AM251, *n* = 8 for normal + AM251, *n* = 9 for CCI + WIN55, 212‐2, *n* = 9 for sham CCI + WIN55, 212‐2, and *n* = 7 for normal + WIN55, 212‐2. Protein expression levels were normalized to GAPDH. Group comparisons were performed using one‐way ANOVA followed by Tukey–Kramer post hoc test. ^*^ *P* < 0.05, CCI group vs the sham group(+A, +W), normal group(+A, +W), ^★^ *P* < 0.01, CCI+A group vs the sham+A(CCI, +W) and normal+A(CCI, +W) groups. ^※^ *P* < 0.01, CCI+W group vs sham+W(CCI, +A) and normal+W(CCI, +A) groups. ^☆^ *P* < 0.01 CCI+A group vs CCI+W group. ^#^ *P* < 0.05, CCI group vs CCI+A or CCI+W group.

**FIGURE 6:**
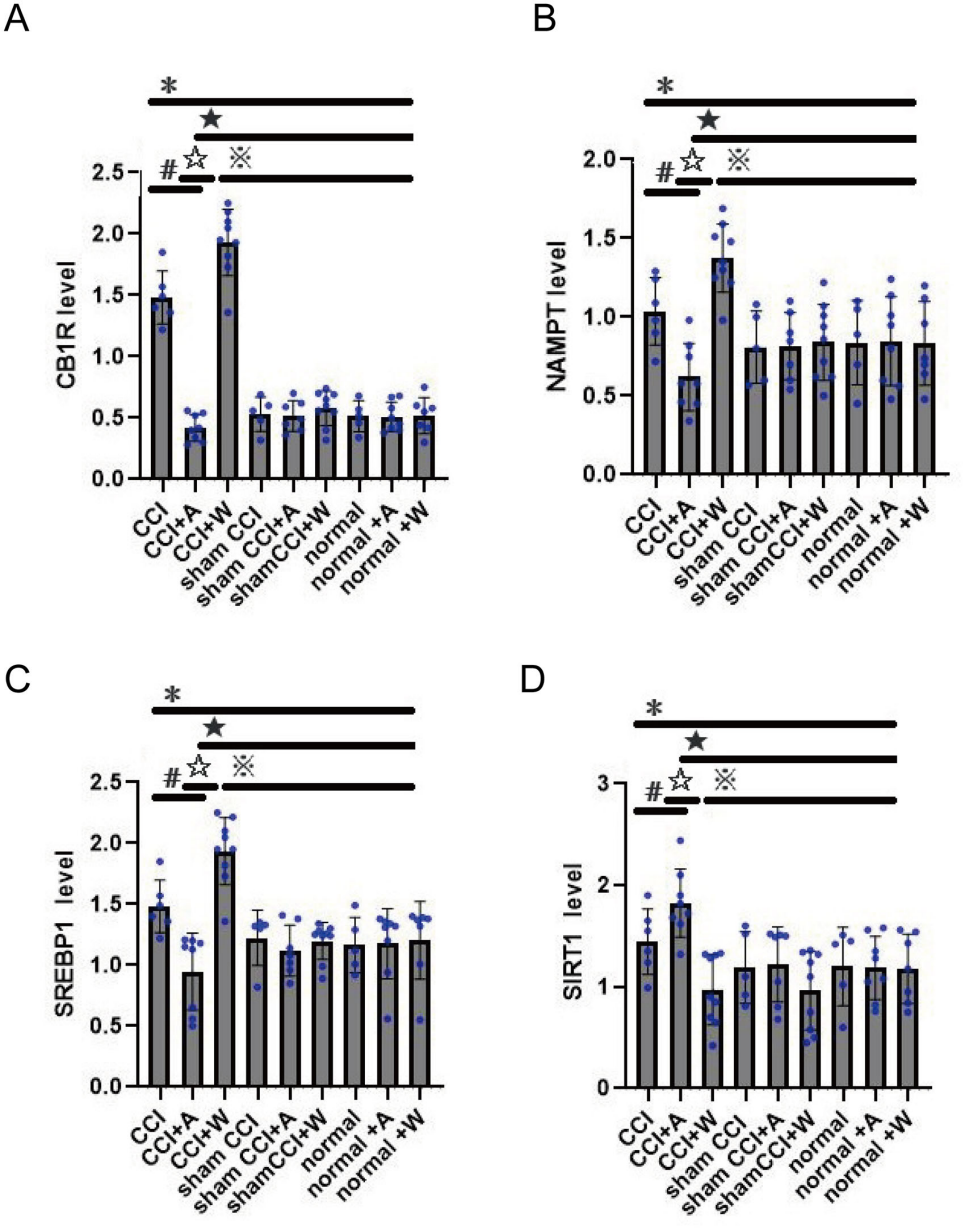
mRNA expression levels of metabolic regulators in the spinal dorsal horn. Quantitative PCR (qPCR) analysis showing the relative mRNA transcription levels of (A) CB1R, (B) NAMPT, (C) SREBP1, and (D) SIRT1. Expression levels were normalized to *β*‐actin. Data are presented as mean ± SD. *n* = 8 for CCI + AM251, *n* = 7 for sham CCI + AM251, *n* = 8 for normal + AM251, *n* = 9 for CCI + WIN55, 212‐2, *n* = 9 for sham CCI + WIN55, 212‐2, and *n* = 7 for normal + WIN55, 212‐2. Relative mRNA expression was analyzed using the 2^−ΔΔCt^ method. Group comparisons were performed using one‐way ANOVA followed by Tukey–Kramer post hoc test. ^*^ *P* < 0.05, CCI group vs the sham group(+A, +W), normal group(+A, +W), ^★^ *P* < 0.01, CCI+A group vs the sham+A(CCI, +W) and normal+A(CCI, +W) groups. ^※^ *P* < 0.01, CCI+W group vs sham+W(CCI, +A) and normal+W(CCI, +A) groups. ^☆^ *P* < 0.01 CCI+A group vs CCI+W group. ^#^ *P* < 0.05, CCI group vs CCI+A or CCI+W group.

**TABLE 1:** Grayscale values for CB1R, NAMPT, SREBP1, and SIRT1 across groups.

	CCI	Sham CCI	Normal	CCI+A	ShamCCI+A	Normal+A	CCI+W	Sham CCI+W	normal+W
CB1R	1.02 ± 0.23^*#^	0.88 ± 0.25	0.92 ± 0.27	0.52 ± 0.25^★☆^	0.84 ± 0.23	0.88 ± 0.26	1.27 ± 0.26^※^	0.89 ± 0.23	0.91 ± 0.26
SREBP1	1.35 ± 0.24^*#^	1.12 ± 0.27	1.14 ± 0.26	0.84 ± 0.23^★☆^	1.16 ± 0.25	1.15 ± 0.24	1.67 ± 0.25^※^	1.15 ± 0.22	1.14 ± 0.25
NAMPT	1.74 ± 0.26^*#^	1.44 ± 0.28	1.41 ± 0.23	0.74 ± 0.23^★☆^	1.43 ± 0.27	1.41 ± 0.26	2.04 ± 0.26^※^	1.46 ± 0.26	1.42 ± 0.23
SIRT1	1.91 ± 0.38^*#^	1.74 ± 0.32	1.72 ± 0.35	2.25 ± 039^★☆^	1.75 ± 0.34	1.73 ± 037	1.36 ± 0.34^※^	1.78 ± 0.38	1.73 ± 0.36

*Note*: Data are presented as mean ± SD. *n* = 6 for CCI, *n* = 5 for sham CCI, *n* = 5 for normal control, *n* = 8 for CCI + AM251, *n* = 7 for sham CCI + AM251, *n* = 8 for normal + AM251, *n* = 9 for CCI + WIN55, 212‐2, *n* = 9 for sham CCI + WIN55, 212‐2, and *n* = 7 for normal + WIN55, 212‐2. Protein expression levels were quantified by grayscale analysis of Western blot bands and normalized to GAPDH. Comparisons among groups were performed using one‐way ANOVA followed by Tukey–Kramer post hoc test. ^*^ *P* < 0.05, indicates a significant difference between the CCI group and the sham group(+A, +W), normal group(+A, +W), ^★^ *P* < 0.01 indicates a significant difference for the CCI+A group compared to both the sham+A(CCI, +W) and normal+A(CCI, +W) groups. ^※^ *P* < 0.01 signifies a significant difference for the CCI+W group when compared to the sham+W(CCI, +A) and normal+W(CCI, +A) groups. ^☆^ *P* < 0.01 denotes a significant difference between the CCI+A and CCI+W groups.^#^ *P* < 0.05 denotes a significant difference between the CCI group and the CCI+A or CCI+W groups.

**TABLE 2:** Relative transcription levels of CB1R, NAMPT, SREBP1, and SIRT1 in the lumbar spinal cord dorsal horn across groups.

	CCI	Sham CCI	Normal	CCI+A	ShamCCI+A	Normal+A	CCI+W	ShamCCI +W	Normal+W
CB1R	0.71 ± 0.14^*#^	0.53 ± 0.14	0.51 ± 0.13	0.42 ± 0.11^★☆^	0.52 ± 0.13	0.51 ± 0.12	1.02 ± 0.15^※^	0.58 ± 0.14	0.52 ± 0.15
SREBP1	1.48 ± 0.22^*#^	1.14 ± 0.28	1.2 ± 0.23	0.9 ± 0.32^★☆^	1.12 ± 0.21	1.18 ± 0.23	1.93 ± 0.28^※^	1.21 ± 0.3	1.19 ± 0.32
NAMPT	1.04 ± 0.23^*#^	0.81 ± 0.25	0.84 ± 0.27	0.62 ± 0.22^★☆^	0.82 ± 0.21	0.85 ± 0.28	1.37 ± 0.22^※^	0.84 ± 0.24	0.83 ± 0.27
SIRT1	1.45 ± 0.32^*#^	1.19 ± 0.34	1.21 ± 0.35	1.81 ± 0.36^★☆^	1.22 ± 0.37	1.19 ± 0.30	0.96 ± 0.34^※^	1.15 ± 0.37	1.18 ± 0.34

*Note*: Data are presented as mean ± SD. *n* = 6 for CCI, *n* = 5 for sham CCI, *n* = 5 for normal control, *n* = 8 for CCI + AM251, *n* = 7 for sham CCI + AM251, *n* = 8 for normal + AM251, *n* = 9 for CCI + WIN55, 212‐2, *n* = 9 for sham CCI + WIN55, 212‐2, and *n* = 7 for normal + WIN55, 212‐2. Relative mRNA expression levels were calculated using the 2^−ΔΔCt^ method. Comparisons among groups were performed using one‐way ANOVA followed by Tukey–Kramer post hoc test. ^*^ *P* < 0.05, indicates a significant difference between the CCI group and the sham group(+A, +W), normal group(+A, +W), ^★^ *P* < 0.01 indicates a significant difference for the CCI+A group compared to both the sham+A(CCI, +W) and normal+A(CCI, +W) groups. ^※^ *P* < 0.01 signifies a significant difference for the CCI+W group when compared to the sham+W(CCI, +A) and normal+W(CCI, +A) groups. ^☆^ *P* < 0.01 denotes a significant difference between the CCI+A and CCI+W groups. ^#^ *P* < 0.05 denotes a significant difference between the CCI group and the CCI+A or CCI+W groups.

## 4 Discussion

Neuropathic pain involves maladaptive plasticity within spinal nociceptive circuits, and cannabinoid CB1 receptor (CB1R) signaling has emerged as an important but incompletely defined modulator of these processes (Colloca et al. 2017; Latremoliere and Woolf 2009; Anthony et al. 2020; Campos et al. 2021; Milligan et al. 2019). In this study, we investigated the association between CB1R modulation, synaptic plasticity, and pain‐related behaviors in a mouse model of neuropathic pain. We found that pharmacological activation of CB1R was accompanied by reduced mechanical and thermal hypersensitivity and attenuation of C‐fiber–evoked LTP in the SDH. This behavioral pattern is consistent with previous studies showing that WIN55,212‐2 alleviates thermal and mechanical hyperalgesia or allodynia in rat CCI models of neuropathic pain (Herzberg et al. 1997; Costa et al. 2003). These changes occurred in parallel with coordinated alterations in systemic lipid indices and in the expression of metabolic regulators, including SREBP1, NAMPT, and SIRT1, in the spinal cord. Together, these findings suggest that CB1R signaling is associated with a metabolic regulatory network that may influence spinal synaptic plasticity during neuropathic pain.

Mechanistically, CB1R is a Gi/o protein‐coupled receptor that classically suppresses presynaptic neurotransmitter release. Upon activation, CB1R inhibits adenylyl cyclase activity and reduces intracellular cAMP signaling. CB1R activation can also inhibit presynaptic voltage‐gated Ca^2^^+^ channels and limit Ca^2^^+^ influx into nerve terminals, thereby reducing the release of excitatory neurotransmitters such as glutamate. In the SDH, such presynaptic inhibition may decrease excitatory nociceptive transmission and weaken the induction or maintenance of activity‐dependent LTP. This canonical Gi/o‐mediated mechanism provides a plausible explanation for the reduced C‐fiber–evoked potentiation observed after WIN55,212‐2 treatment in the present study.

The SDH is a critical site for nociceptive signal processing, and activity‐dependent synaptic plasticity, particularly LTP, is widely regarded as a cellular substrate of central sensitization (Latremoliere and Woolf 2009; Chen and Tang 2024). In this context, our electrophysiological data show that CB1R activation was associated with attenuation of exaggerated C‐fiber LTP induced by CCI. These findings are consistent with previous reports demonstrating that endocannabinoid signaling suppresses excitatory synaptic transmission in the central nervous system (Zou and Kumar 2018; Martinez Ramirez et al. 2023), and with evidence that dorsal horn structure/function is reshaped after peripheral nerve injury (Kurabe et al. 2022). Rather than directly proving a causal mechanism, our results support the notion that CB1R modulation is linked to reduced spinal synaptic hyperexcitability, which may contribute to the observed improvement in pain‐related behaviors.

An important aspect of this study is the observed association between CB1R signaling and SREBP1 expression in the SDH under neuropathic conditions. SREBP1 is a master transcription factor governing lipid biosynthesis and has been implicated in neuronal activity–dependent processes (Chen et al. 2017). Neuronal membranes and synaptic structures are highly sensitive to lipid composition, as lipids constitute essential components of cell membranes, synaptic vesicles, and lipid rafts that organize receptor signaling (Grassi et al. 2020; Tsui‐Pierchala et al. 2002). In our model, CB1R inhibition was associated with reduced SREBP1 expression, whereas CB1R activation was associated with increased SREBP1 expression in the SDH. These findings suggest that CB1R modulation is accompanied by changes in lipid regulatory programs that may be permissive for altered synaptic function, although direct effects on membrane lipid composition were not assessed in this study. Given that CB1R signaling can be organized within lipid rafts and exhibits subsynaptic raft targeting, this provides a plausible membrane‐level context for linking CB1R activity to lipid‐dependent signaling organization (Bari et al. 2005; Saumell‐Esnaola et al. 2021).

An additional observation requiring cautious interpretation is the reduction of CB1R expression in the SDH after AM251 treatment. Although AM251 functions as a CB1R antagonist/inverse agonist, sustained receptor blockade may induce adaptive changes in receptor trafficking, turnover, or internalization (Hsieh et al. 1999; Coutts et al. 2001). Alternatively, prolonged CB1R inhibition may alter the overall activity state of dorsal horn neurons, leading to feedback suppression of CB1R expression (Martín‐García et al. 2009). Thus, the reduced CB1R level observed in the CCI + AM251 group may reflect a secondary adaptive response rather than a simple direct effect of AM251 on CB1R expression. Additional time‐course and cell‐type‐specific studies will be required to clarify this mechanism.

In addition, we observed coordinated changes in the NAMPT/SIRT1 pathway following CB1R modulation. NAMPT is the rate‐limiting enzyme in NAD^+^ biosynthesis and plays a central role in maintaining cellular energy balance and redox homeostasis (Zhang et al. 2025). SIRT1, an NAD^+^‐dependent deacetylase, functions as a metabolic sensor and has been implicated in neuronal stress responses and synaptic regulation (Michan et al. 2010). In the present study, CB1R inhibition was associated with reduced NAMPT expression and increased SIRT1 expression, whereas CB1R activation showed the opposite pattern. These observations indicate that CB1R signaling is linked to shifts in metabolic stress–response pathways in the spinal cord during neuropathic pain, although the functional consequences of these changes remain to be fully elucidated.

C‐fiber–evoked spinal LTP is closely linked to glutamatergic transmission, NMDA receptor activation, and Ca^2^^+^ influx (Liu and Sandkühler 1995; Liu and Zhou 2015). Therefore, the metabolic changes observed in the present study may influence LTP by converging on these classical synaptic mechanisms. NAMPT regulates NAD^+^ availability and SIRT1‐dependent metabolic signaling, which may affect neuronal redox state, NMDA receptor phosphorylation or expression, and downstream Ca^2^^+^‐dependent plasticity pathways. Thus, CB1R‐associated regulation of the NAMPT/SIRT1 axis may contribute to altered C‐fiber LTP by modulating glutamatergic/NMDA receptor‐dependent signaling. Nevertheless, this possibility remains to be directly tested, as NMDA receptor phosphorylation, receptor subunit expression, and Ca^2^^+^ dynamics were not assessed in the current study.

We also detected alterations in circulating lipid indices following CB1R modulation, but these findings should be interpreted cautiously. In the present study, clear lipid changes were mainly observed under CCI conditions, whereas AM251 or WIN55,212‐2 did not produce consistent changes in sham‐operated or normal control mice. This suggests that the systemic lipid response to CB1R modulation may depend on the neuropathic pain state rather than reflecting a uniform drug effect under basal conditions. Neuropathic pain is accompanied by sustained stress responses, altered autonomic activity, neuroimmune activation, and changes in energy metabolism, all of which may interact with CB1R signaling and modify lipid handling (Wang et al. 2025; Vyverman et al. 2026). Therefore, the lipid profile observed in CCI mice may represent a combined effect of CB1R modulation and injury‐related metabolic adaptation.

This context‐dependent interpretation may explain the discrepancy between our lipid findings and previous metabolic studies. For example, van Eenige et al. reported that CB1R inverse agonism reduced triglycerides and increased HDL‐C in APOE*3‐Leiden.CETP mice, a metabolic disease model of dyslipidemia and atherosclerosis (van Eenige et al. 2021). In contrast, our study used a neuropathic pain model in KM mice with short‐term pharmacological intervention. Differences in disease background, animal strain, drug exposure, nutritional/metabolic status, and pain‐related stress responses may therefore contribute to the divergent lipid profiles (Fiore et al. 2023). Thus, our data should not be interpreted as evidence that CB1R activation or inhibition uniformly regulates lipoprotein metabolism, but rather as evidence that CB1R modulation is associated with systemic lipid remodeling under neuropathic injury conditions.

Neuroinflammation may also link CB1R‐associated metabolic regulation to spinal synaptic plasticity. In neuropathic pain, spinal glial activation and cytokines such as TNF‐α and IL‐1β can enhance excitatory transmission, facilitate NMDA receptor‐dependent signaling, and promote C‐fiber LTP (Gruber‐Schoffnegger et al. 2013; Zhou et al. 2019). Therefore, the CB1R/SREBP1‐related metabolic changes observed here may influence synaptic plasticity not only through lipid raft‐dependent receptor organization, but also through modulation of the inflammatory microenvironment in the SDH. Since inflammatory markers were not examined in this study, this possibility remains to be tested in future work.

Nevertheless, several limitations of the present study should be acknowledged. Our conclusions are primarily based on pharmacological modulation. It is well‐documented that AM251 is not an absolutely selective CB1R antagonist/inverse agonist and may exhibit off‐target effects, while WIN55,212‐2 can also activate CB2R at certain concentrations. Therefore, these findings should be interpreted as associations rather than definitive proof of a CB1R‐specific causal cascade. Future studies utilizing genetic CB1R knockout models and selective CB2R controls will be essential to establish receptor specificity.

In conclusion, our findings indicate that CB1R modulation is associated with coordinated alterations in SDH synaptic plasticity, pain behaviors, and the SREBP1/NAMPT/SIRT1 metabolic framework. This work identifies a metabolic link to cannabinoid‐mediated pain modulation and provides a basis for future mechanistic investigations. Genetic knockout models and selective small‐molecule modulators targeting this pathway will be needed to clarify causal mechanisms and explore mechanism‐based analgesic strategies.

## Author Contributions

**Lu Liu** and **Chenglong Wu** carried out the studies, participated in collecting data, and drafted the manuscript. **Hui Xia** and **Jie Cheng** performed the statistical analysis and participated in its design. **Liang Zhong** and **Jiang Wu** participated in the acquisition, analysis, or interpretation of data and drafted the manuscript. All authors read and approved the final manuscript.

## Funding

The authors have nothing to report.

## Ethics Statement

This study was carried out in accordance with the principles of the Basel Declaration. All animal studies (including the mice euthanasia procedure) were conducted according to the AAALAC and the IACUC guidelines and approved by Wuhan University Animal Laboratory Center with the ethics approval number AUP2019073.

## Consent

The authors have nothing to report.

## Conflicts of Interest

The authors declare no conflicts of interest.

## Supporting information




**Supplementary Material**: brb371837‐sup‐0001‐SuppMat.docx

**Supplementary Tables**: brb371837‐sup‐0002‐TablesS1‐S8.docx

## Data Availability

All data generated or analyzed during this study are included in this published article and its supplementary information files.

## References

[brb371837-bib-0001] Anthony, A. T. , S. Rahmat , P. Sangle , O. Sandhu , and S. Khan . 2020. “Cannabinoid Receptors and Their Relationship With Chronic Pain: A Narrative Review.” Cureus 12, no. 9: e10436. 10.7759/cureus.10436 PMC7557112 33072446

[brb371837-bib-0002] Bari, M. , N. Battista , F. Fezza , A. Finazzi‐Agrò , and M. Maccarrone . 2005. “Lipid Rafts Control Signaling of Type‐1 Cannabinoid Receptors in Neuronal Cells.” Journal of Biological Chemistry 280, no. 13: 12212–12220. 10.1074/jbc.M411642200. 15657045

[brb371837-bib-0003] Cajiao‐Manrique, M. D. M. , R. Maldonado , and E. Martín‐García . 2023. “A Male Mouse Model of WIN 55,212–2 Self‐Administration to Study Cannabinoid Addiction.” Frontiers In Pharmacology 14: 1143365. 10.3389/fphar.2023.1143365. PMC10083303 37050910

[brb371837-bib-0004] Campos, R. M. P. , A. F. L. Aguiar , Y. Paes‐Colli , et al. 2021. “Cannabinoid Therapeutics in Chronic Neuropathic Pain: From Animal Research to Human Treatment.” Frontiers in Physiology 12: 785176. 10.3389/fphys.2021.785176. PMC8669747 34916962

[brb371837-bib-0005] Cavalli, E. , S. Mammana , F. Nicoletti , P. Bramanti , and E. Mazzon . 2019. “The Neuropathic Pain: An Overview of the Current Treatment and Future Therapeutic Approaches.” International Journal of Immunopathology and Pharmacology 33: 1–10. 10.1177/2058738419838383. PMC6431761 30900486

[brb371837-bib-0006] Charan, J. , and N. D. Kantharia . 2013. “How to Calculate Sample Size in Animal Studies?” Journal of Pharmacology and Pharmacotherapeutics 4, no. 4: 303–306. 10.4103/0976-500X.119726. PMC3826013 24250214

[brb371837-bib-0007] Chen, X. , and S. J. Tang . 2024. “Neural Circuitry Polarization in the Spinal Dorsal Horn (SDH): A Novel Form of Dysregulated Circuitry Plasticity During Pain Pathogenesis.” Cells 13, no. 5: 398. 10.3390/cells13050398. PMC10930392 38474361

[brb371837-bib-0008] Chen, Y. , S. Bang , M. F. McMullen , et al. 2017. “Neuronal Activity‐Induced Sterol Regulatory Element Binding Protein‐1 (SREBP1) is Disrupted in Dysbindin‐Null Mice—Potential Link to Cognitive Impairment in Schizophrenia.” Molecular Neurobiology 54, no. 3: 1699–1709. 10.1007/s12035-016-9773-x. PMC4982840 26873854

[brb371837-bib-0009] Chesworth, R. , G. Watt , J. Howard , G. Ding , and T. Karl . 2026. “Genetic Risk for Schizophrenia Modulates WIN 55,212‐2 Reward: Evidence from a Heterozygous Transmembrane Domain Neuregulin 1 Mutant Mouse Model.” *Psychopharmacology*: 1–14. 10.1007/s00213-026-07080-1. 42067687

[brb371837-bib-0010] Colloca, L. , T. Ludman , D. Bouhassira , et al. 2017. “Neuropathic Pain.” Nature Reviews Disease Primers 3: 17002. 10.1038/nrdp.2017.2. PMC5371025 28205574

[brb371837-bib-0011] Costa, B. , M. Colleoni , S. Conti , et al. 2003. “Repeated Treatment With the Synthetic Cannabinoid WIN 55,212‐2 Reduces Both Hyperalgesia and Production of Pronociceptive Mediators in a Rat Model of Neuropathic Pain.” British Journal of Pharmacology 141, no. 1: 4–8. 10.1038/sj.bjp.0705587. PMC1574173 14662732

[brb371837-bib-0012] Coutts, A. A. , S. Anavi‐Goffer , R. A. Ross , et al. 2001. “Agonist‐Induced Internalization and Trafficking of Cannabinoid CB_1_ Receptors in Hippocampal Neurons.” The Journal of Neuroscience 21, no. 7: 2425–2433. 10.1523/JNEUROSCI.21-07-02425.2001. PMC6762401 11264316

[brb371837-bib-0013] Fiore, N. T. , S. R. Debs , J. P. Hayes , S. S. Duffy , and G. Moalem‐Taylor . 2023. “Pain‐Resolving Immune Mechanisms in Neuropathic Pain.” Nature Reviews Neurology 19, no. 4: 199–220. 10.1038/s41582-023-00777-3 36859719

[brb371837-bib-0014] Grassi, S. , P. Giussani , L. Mauri , S. Prioni , S. Sonnino , and A. Prinetti . 2020. “Lipid Rafts and Neurodegeneration: Structural and Functional Roles in Physiologic Aging and Neurodegenerative Diseases.” Journal of Lipid Research 61, no. 5: 636–654. 10.1194/jlr.TR119000427. PMC7193971 31871065

[brb371837-bib-0015] Gruber‐Schoffnegger, D. , R. Drdla‐Schutting , C. Hönigsperger , G. Wunderbaldinger , M. Gassner , and J. Sandkühler . 2013. “Induction of Thermal Hyperalgesia and Synaptic Long‐Term Potentiation in the Spinal Cord Lamina I by TNF‐α and IL‐1β Is Mediated by Glial Cells.” The Journal of Neuroscience 33, no. 15: 6540–6551. 10.1523/JNEUROSCI.5087-12.2013. PMC6619063 23575851

[brb371837-bib-0016] Herzberg, U. , E. Eliav , G. J. Bennett , and I. J. Kopin . 1997. “The Analgesic Effects of R(+)‐WIN 55,212–2 Mesylate, a High Affinity Cannabinoid Agonist, in a Rat Model of Neuropathic Pain.” Neuroscience Letters 221, no. 2–3: 157–160. 10.1016/S0304-3940(96)13308-5. 9121688

[brb371837-bib-0017] Hsieh, C. , S. Brown , C. Derleth , and K. Mackie . 1999. “Internalization and Recycling of the CB1 Cannabinoid Receptor.” Journal of Neurochemistry 73, no. 2: 493–501. 10.1046/j.1471-4159.1999.0730493.x. 10428044

[brb371837-bib-0018] Kim, J. S. , C. S. Yoon , and D. R. Park . 2014. “NAMPT Regulates Mitochondria Biogenesis via NAD Metabolism and Calcium Binding Proteins During Skeletal Muscle Contraction.” Journal of Exercise Nutrition and Biochemistry 18, no. 3: 259–266. 10.5717/jenb.2014.18.3.259. PMC4241898 25566462

[brb371837-bib-0019] Kurabe, M. , M. Sasaki , K. Furutani , H. Furue , Y. Kamiya , and H. Baba . 2022. “Structural and Functional Properties of Spinal Dorsal Horn Neurons After Peripheral Nerve Injury Change Overtime via Astrocyte Activation.” iScience 25, no. 12: 105555. 10.1016/j.isci.2022.105555. PMC9700017 36444301

[brb371837-bib-0020] Latremoliere, A. , and C. J. Woolf . 2009. “Central Sensitization: A Generator of Pain Hypersensitivity by Central Neural Plasticity.” The Journal of Pain 10, no. 9: 895–926. 10.1016/j.jpain.2009.06.012. PMC2750819 19712899

[brb371837-bib-0021] Liu, X. G. , and J. Sandkühler . 1995. “Long‐Term Potentiation of C‐Fiber‐Evoked Potentials in the Rat Spinal Dorsal Horn is Prevented by Spinal N‐Methyl‐D‐Aspartic Acid Receptor Blockage.” Neuroscience Letters 191, no. 1–2: 43–46. 10.1016/0304-3940(95)11553-0. 7659287

[brb371837-bib-0022] Liu, X.‐G. , and L.‐J. Zhou . 2015. “Long‐Term Potentiation at Spinal C‐Fiber Synapses: A Target for Pathological Pain.” Current Pharmaceutical Design 21, no. 7: 895–905. 10.2174/1381612820666141027115949. 25345608

[brb371837-bib-0023] Martinez Ramirez, C. E. , G. Ruiz‐Pérez , T. M. Stollenwerk , C. Behlke , A. Doherty , and C. J. Hillard . 2023. “Endocannabinoid Signaling in the Central Nervous System.” Glia 71, no. 1: 5–35. 10.1002/glia.24280. PMC10167744 36308424

[brb371837-bib-0024] Martín‐García, E. , A. Burokas , M. Martin , et al. 2009. “Central and Peripheral Consequences of the Chronic Blockade of CB1 Cannabinoid Receptor With Rimonabant or Taranabant.” Journal of Neurochemistry 112, no. 5: 1338–13351. 10.1111/j.1471-4159.2009.06549.x 20028452

[brb371837-bib-0025] Michan, S. , Y. Li , M. M.‐H. Chou , et al. 2010. “SIRT1 is Essential for Normal Cognitive Function and Synaptic Plasticity.” Journal of Neuroscience 30, no. 29: 9695–9707. 10.1523/JNEUROSCI.0027-10.2010. PMC2921958 20660252

[brb371837-bib-0026] Milligan, A. L. , T. A. Szabo‐Pardi , and M. D. Burton . 2019. “Cannabinoid Receptor Type 1 and Its Role as an Analgesic: An Opioid Alternative?” Journal of Dual Diagnosis 16, no. 1: 106–119. 10.1080/15504263.2019.1668100. PMC7007359 31596190

[brb371837-bib-0027] Milligan, A. L. , T. A. Szabo‐Pardi , and M. D. Burton . 2020. “Cannabinoid Receptor Type 1 and Its Role as an Analgesic: An Opioid Alternative?” Journal of Dual Diagnosis 16, no. 1: 106–119. 10.1080/15504263.2019.1668100. PMC7007359 31596190

[brb371837-bib-0028] O'Keefe, L. , T. Vu , A. C. Simcocks , et al. 2022. “CB1 Ligand AM251 Induces Weight Loss and Fat Reduction in Addition to Increased Systemic Inflammation in Diet‐Induced Obesity.” International Journal of Molecular Sciences 23, no. 19: 11447. 10.3390/ijms231911447. PMC9569643 36232744

[brb371837-bib-0029] Ostadhadi, S. , A. Haj‐Mirzaian , V. Nikoui , N. Kordjazy , and A.‐R. Dehpour . 2016. “Involvement of Opioid System in Antidepressant‐Like Effect of the Cannabinoid C_1_ Receptor Inverse Agonist AM ‐251 After Physical Stress in Mice.” Clinical and Experimental Pharmacology and Physiology 43, no. 2: 203–212. 10.1111/1440-1681.12518. 26609670

[brb371837-bib-0030] Sandkühler, J. , and X. Liu . 1998. “Induction of Long‐Term Potentiation at Spinal Synapses by Noxious Stimulation or Nerve Injury.” European Journal of Neuroscience 10, no. 7: 2476–2480. 10.1046/j.1460-9568.1998.00278.x. 9749775

[brb371837-bib-0031] Saumell‐Esnaola, M. , S. Barrondo , G. García del Caño , et al. 2021. “Subsynaptic Distribution, Lipid Raft Targeting and G Protein‐Dependent Signalling of the Type 1 Cannabinoid Receptor in Synaptosomes from the Mouse Hippocampus and Frontal Cortex.” Molecules 26, no. 22: 6897. 10.3390/molecules26226897. PMC8621520 34833992

[brb371837-bib-0032] Tsui‐Pierchala, B. A. , M. Encinas , J. Milbrandt , and E. M. Johnson . 2002. “Lipid Rafts in Neuronal Signaling and Function.” Trends in Neurosciences 25, no. 8: 412–417. 10.1016/S0166-2236(02)02215-4. 12127758

[brb371837-bib-0033] Ueda, M. , H. Iwasaki , S. Wang , et al. 2014. “Cannabinoid Receptor Type 1 Antagonist, AM251, Attenuates Mechanical Allodynia and Thermal Hyperalgesia After Burn Injury.” Anesthesiology 121, no. 6: 1311–1319. 10.1097/ALN.0000000000000422. PMC4237656 25188001

[brb371837-bib-0034] van Eenige, R. , Z. Ying , L. Tambyrajah , et al. 2021. “Cannabinoid Type 1 Receptor Inverse Agonism Attenuates Dyslipidemia and Atherosclerosis in APOE∗3‐Leiden.CETP Mice.” Journal of Lipid Research 62: 100070. 10.1016/j.jlr.2021.100070 PMC8082266 33766515

[brb371837-bib-0035] Vyverman, J. , R. De Baere , I. Timmers , I. Coppieters , J. Van Oosterwijck , and M. Moerkerke . 2026. “The Stress–Pain Connection in Chronic Primary Pain: A Systematic Review and Meta‐Analysis of Physiological Stress Markers in Relation to Experimental Pain Responses.” Neuroscience & Biobehavioral Reviews 184: 106604. 10.1016/j.neubiorev.2026.106604. 41679412

[brb371837-bib-0036] Wang, J. , B. Liu , J. Liu , et al. 2025. “The Crosstalk Between Brain Energy Metabolism and Neuropathic Pain: Mechanisms and Therapeutic Implications.” Metabolites 15, no. 12: 755. 10.3390/metabo15120755. PMC12735086 41440997

[brb371837-bib-0037] Zhang, W. , H. Ren , W. Chen , et al. 2025. “Nicotinamide Phosphoribosyltransferase in NAD+ Metabolism: Physiological and Pathophysiological Implications.” Cell Death Discovery 11, no. 1: 371. 10.1038/s41420-025-02672-w. PMC12332177 40775221

[brb371837-bib-0038] Zhou, L.‐J. , J. Peng , Y.‐N. Xu , et al. 2019. “Microglia are Indispensable for Synaptic Plasticity in the Spinal Dorsal Horn and Chronic Pain.” Cell Reports 27, no. 13: 3844–3859. 10.1016/j.celrep.2019.05.087. PMC7060767 31242418

[brb371837-bib-0039] Zou, S. , and U. Kumar . 2018. “Cannabinoid Receptors and the Endocannabinoid System: Signaling and Function in the Central Nervous System.” International Journal of Molecular Sciences 19, no. 3: 833. 10.3390/ijms19030833. PMC5877694 29533978

