# Supplementary material for: Cannabinoid CB1 Receptor Regulates Spinal Dorsal Horn Synaptic Plasticity in a Mouse Model of Neuropathic Pain through the CB1R/SREBP1/NAMPT/SIRT1 Metabolic Regulatory Framework
Source: Brain Behav. 2026 Oct 13;16(10):e71837. doi: 10.1002/brb3.71837 (PMC13659934; doi:10.1002/brb3.71837)
Supplement: Supplementary file 1 [file BRB3-16-e71837-s001.docx]

**Materials and Methods**

**Animals**

A total of 68 male KM mice, 6 weeks of age and weighing 20–25 g, were obtained from Beijing Unilever Company. Only male mice were used in this study to minimize potential variability associated with estrous cycle-related hormonal fluctuations, which may influence pain sensitivity and endogenous cannabinoid signaling. All animals were housed under controlled conditions with a temperature of 20 ± 1°C, relative humidity of 50 ± 1%, and a 12 h/12 h light/dark cycle, with free access to food and water. All animal experiments, including euthanasia procedures, were conducted in accordance with AAALAC and IACUC guidelines and were approved by the Wuhan University Animal Laboratory Center under ethics approval number AUP2019073.

**Mouse CCI model and grouping**

The mouse model of chronic constriction injury (CCI) of the sciatic nerve was established as previously described, with minor modifications. Briefly, mice were anesthetized with an intraperitoneal injection of 1% sodium pentobarbital at 35–40 mg/kg. After routine skin preparation and disinfection, a 0.8–1.0 cm incision was made on the lateral surface of the left thigh. The biceps femoris muscle was bluntly separated to expose the left sciatic nerve. Three loose ligatures were placed around the sciatic nerve using 4-0 or 5-0 chromic catgut, with an interval of approximately 1 mm between adjacent ligatures. Each ligature was tied until a slight constriction of the nerve was observed, corresponding to approximately one-third reduction in nerve diameter, and a brief twitch of the ipsilateral toes could be seen. Care was taken to avoid excessive compression, impaired epineural blood flow, or obvious purple discoloration of the nerve. The muscle and skin were then closed in layers.

Successful model establishment was evaluated by behavioral changes after surgery. Mechanical and thermal hypersensitivity became stable approximately 7 days after CCI and persisted for more than 14 days. Mice showing stable pain hypersensitivity after surgery were considered successfully modeled and were included in subsequent experiments.

The 68 mice were randomly divided into nine groups: normal control group, n = 5; CCI group, n = 8; sham group, n = 7; CCI + AM251 group, n = 8; sham + AM251 group, n = 7; normal control + AM251 group, n = 8; CCI + WIN55,212-2 group, n = 9; sham + WIN55,212-2 group, n = 9; and normal control + WIN55,212-2 group, n = 7. AM251, a CB1R antagonist, was administered intraperitoneally at 5 mg/kg twice daily from day 11 to day 13 after modeling. WIN55,212-2, a CB1R agonist, was administered intraperitoneally at 1 mg/kg three times daily from day 11 to day 13 after modeling. Behavioral tests were performed on day 7 after surgery to confirm model establishment and again on day 14 after surgery to evaluate drug effects. Electrophysiological recordings were then performed after behavioral testing. After completion of the experiments, plasma lipid indices and spinal dorsal horn tissues were collected for subsequent biochemical and molecular analyses.

**Assessment of mechanical and thermal hypersensitivity**

Mechanical hypersensitivity was assessed by paw withdrawal frequency using von Frey filaments. Mice were individually placed on a metal wire mesh floor in a plastic chamber measuring 20 × 25 × 15 cm and allowed to acclimate for 45 min before testing. The plantar surface of the ipsilateral hind paw was vertically stimulated with von Frey filaments of 0.31, 0.38, 0.45, 0.6, 1.0, 1.4, 2.0, and 4.0 g. Each stimulation lasted 4–5 s. Each filament was applied three times per animal, with an interval of at least 2 min between stimulations. A positive response was defined as rapid paw withdrawal, licking, or flinching immediately after filament application. Paw withdrawal frequency was calculated as follows: number of positive withdrawal responses / 3 × 100%.

Paw withdrawal frequency was used instead of the up-down threshold method because it allows graded assessment of mechanical hypersensitivity across multiple filament forces and is suitable for detecting changes in response probability in mouse neuropathic pain models. This approach reduces reliance on a single estimated threshold value and has been used previously to evaluate mechanical hypersensitivity after peripheral nerve injury.

Thermal hypersensitivity was assessed by paw withdrawal latency. Mice were individually placed in a plastic chamber measuring 7 × 9 × 11 cm on a smooth glass plate with a thickness of 1 mm and were allowed to acclimate for 45 min. A heat source maintained at 55.0 ± 0.5°C was positioned beneath the glass and focused on the plantar surface of the ipsilateral hind paw. A cutoff time of 20 s was used to prevent tissue damage. Each animal was tested three times, with an interval of at least 2 min between tests, and the mean value was used for analysis.

**In Vivo Electrophysiological Recordings**

Electrophysiological recordings were performed on day 14 after CCI, or at the corresponding experimental time point in sham-operated and normal control mice, 24 h after the last drug administration and after completion of behavioral testing. For each animal, only one in vivo electrophysiological recording session was performed after the final PWT and TPT assessments. During this session, baseline evoked field potentials were first recorded, followed by a single HFS conditioning protocol and one post-HFS recording period. A- and C-fiber–evoked components were obtained from the same sciatic nerve stimulation–evoked field potential and were distinguished according to their conduction latencies. Mice were anesthetized with 1% sodium pentobarbital at 35–40 mg/kg by intraperitoneal injection and routinely intubated. Vecuronium bromide at 0.05–0.1 mg/kg was administered intraperitoneally for muscle relaxation, and respiration was supported using a small-animal ventilator at 90–120 breaths/min. Body temperature was maintained at 37°C throughout the recording.

After fixation in a stereotaxic frame, a laminectomy was performed at the T13–L1 vertebral level to expose the lumbar spinal cord. The dura was carefully opened under magnification, and the exposed spinal cord was covered with warmed liquid paraffin maintained at 37°C. Extracellular field potentials were recorded from the ipsilateral spinal dorsal horn using a stainless-steel bipolar electrode with an impedance of 0.5–1 MΩ and a tip diameter of 0.1 mm. The recording electrode was inserted to a depth of approximately 0.1 mm, corresponding approximately to laminae I–II of the spinal dorsal horn. A bipolar stimulating electrode was placed on the left sciatic nerve proximal to the ligation site, and the exposed nerve was kept moist with warmed liquid paraffin throughout the recording.

Baseline responses were evoked by single square-wave pulses at 1.5 V, 0.5 ms, and 1 pulse/min for 30 min. LTP was then induced by high-frequency stimulation(HFS) consisting of four trains at 3 V, 0.5 ms, 100 Hz, and 1 s duration, with a 10 s inter-train interval. This HFS protocol was selected based on established in vivo spinal dorsal horn LTP paradigms, in which brief 100 Hz tetanic stimulation delivered in four 1-s trains with 10-s inter-train intervals reliably induces long-lasting potentiation of C-fiber–evoked field potentials and is widely used to model activity-dependent spinal nociceptive plasticity. After tetanic stimulation, single-pulse stimulation at 1.5 V, 0.5 ms, and 1 pulse/min was resumed for 60 min to monitor changes in evoked field potentials. Signals were acquired using an RM6240 multi-channel recording system in EEG mode at a sampling rate of 8 kHz, with a low-pass filter set to 30 Hz and a time constant of 0.02 s. Recordings were performed in a Faraday cage with proper grounding to minimize electrical noise and baseline drift.

A- and C-fiber–evoked responses were distinguished according to their conduction latencies. Responses occurring within 2–5 ms after stimulation were defined as A-fiber–mediated responses, whereas responses occurring within 18–35 ms were defined as C-fiber–mediated responses. LTP was quantified as changes in both peak amplitude and response area, calculated separately for A- and C-fiber components. For each animal, baseline values were defined as the mean value during the 30-min pre-HFS period, and post-HFS values were calculated from the 90-min recording period after tetanic stimulation. Only one baseline dataset and one post-HFS dataset from each animal were used for quantification, and no repeated independent HFS trials from the same animal were included in the statistical analysis. The magnitude of LTP was expressed as the percentage change from baseline using the following formula: [(post-HFS value − baseline value) / baseline value] × 100%. Successful LTP was defined as a sustained increase of more than 20% above baseline in evoked response amplitude and/or response area for at least 30 consecutive minutes after HFS. The magnitude of LTP was expressed as the percentage change from baseline using the following formula: [(post-HFS value − baseline value) / baseline value] × 100%. Successful LTP was defined as a sustained increase of more than 20% above baseline in evoked response amplitude and/or response area for at least 30 consecutive minutes after HFS.

To maintain physiological stability during surgery and recording, mice received 1.0 ml sterile 0.9% saline by intraperitoneal injection immediately after laminectomy. Animals were monitored throughout the recording for signs of dehydration or circulatory instability, including pale sclera, sunken eyes, or reduced skin turgor. When necessary, an additional 0.5–1.0 ml sterile saline was administered approximately 1 h into the recording session.

Recordings were excluded from electrophysiological analysis according to predefined objective criteria, including failed CCI modeling as indicated by the absence of stable mechanical or thermal hypersensitivity after surgery, excessive movement artifacts or electrical noise causing more than 10% fluctuation of the 30-min baseline response, unstable baseline responses before HFS, unintended drug delivery failure, or non-quantifiable evoked potentials. Stable recordings were ensured by performing experiments in a Faraday cage with proper grounding to minimize electrical noise and baseline drift. After exclusion of non-quantifiable electrophysiological recordings, 64 mice were included in the final electrophysiological analysis: CCI group, n = 6; sham group, n = 5; normal control group, n = 5; CCI + AM251 group, n = 8; sham + AM251 group, n = 7; normal control + AM251 group, n = 8; CCI + WIN55,212-2 group, n = 9; sham + WIN55,212-2 group, n = 9; and normal control + WIN55,212-2 group, n = 7. Multiple comparisons were adjusted using the Tukey–Kramer method.

**Quantitative Assay Kit Measurements**

At the end of the experiment, blood samples were collected from the orbital venous plexus after 12 h of fasting, with free access to water. Approximately 0.5–0.6 mL of whole blood was collected from each mouse into additive-free coagulation tubes. Samples were allowed to clot at room temperature for 45–60 min and centrifuged at 3,000 × g for 10 min at 4°C. The upper clear serum fraction was collected carefully, and grossly hemolyzed samples were excluded.

Serum triglycerides (TG), total cholesterol (TC), low-density lipoprotein cholesterol (LDL-C), and high-density lipoprotein cholesterol (HDL-C) were measured using commercial colorimetric assay kits according to the manufacturer’s instructions. TC, HDL-C, and LDL-C were measured using a cholesterol/HDL/LDL assay kit (ab65390, Abcam, Cambridge, UK). TG was measured using a triglyceride assay kit (ab65336, Abcam, Cambridge, UK). TC was detected using the CHOD-PAP enzymatic colorimetric method, TG using the GPO-PAP enzymatic colorimetric method, and HDL-C/LDL-C using selective precipitation followed by enzymatic colorimetric detection. For each assay, 5 μL of serum was added per well in a 96-well plate, and absorbance was measured at 570 nm using a microplate reader. Lipid concentrations were calculated from the corresponding standard curves.

**Immunohistochemistry**

Lumbar spinal cord segments (L3–L5) were dissected, fixed, embedded, sectioned, and washed with PBS. For antigen retrieval, sections were immersed in citrate antigen retrieval buffer (10 mM sodium citrate, pH 6.0) and heated at 95–100°C for 10–15 min, followed by cooling at room temperature for 20 min. Endogenous peroxidase activity was blocked with 3% hydrogen peroxide for 10 min at room temperature. After washing with PBS, sections were blocked with skim milk powder for 30 min to reduce nonspecific staining.

Sections were then incubated overnight at 4°C with the following primary antibodies: anti-CB1R (Abclonal, Cat. No. A1447, 1:1000), anti-SREBP1 (Abclonal, Cat. No. A15586, 1:1000), anti-NAMPT (Abclonal, Cat. No. A0256, 1:1000), and anti-SIRT1 (Proteintech, Cat. No. 13161-1-AP, 1:1000). After washing with PBS, sections were incubated with HRP-conjugated secondary antibodies for 1 h at room temperature. Immunoreactive signals were visualized using 3,3′-diaminobenzidine (DAB) chromogenic detection for 2–5 min under microscopic monitoring, and the reaction was stopped by rinsing with distilled water. Sections were then counterstained with hematoxylin, dehydrated, cleared, and mounted.

Negative control sections were processed in parallel using the same staining procedure, except that the primary antibodies were replaced with PBS. No specific DAB-positive staining was observed in the negative control sections, supporting the specificity of the immunohistochemical staining.

Images were captured under a light microscope from the ipsilateral spinal dorsal horn. Quantitative analysis was restricted to laminae I–II, the superficial dorsal horn region that receives nociceptive afferent input. For each animal, three to five randomly selected fields within laminae I–II were analyzed using ImageJ software. The mean optical density (MOD) of DAB-positive staining was measured after background subtraction, and the averaged MOD value was used to represent the relative expression level of each target protein.

**Western Blot**

Lumbar spinal cord segments (L3–L5) were dissected and homogenized in ice-cold lysis buffer at a ratio of 12.5 μl/mg tissue. The homogenates were centrifuged at 13,000 rpm for 15 min at 4°C, and the supernatants were collected for protein concentration determination using a BCA protein assay. Equal amounts of protein were mixed with loading buffer, denatured at 95°C for 5 min, and separated by SDS-PAGE.

For electrophoresis, protein samples were first run at 80 V for approximately 20–30 min through the stacking gel and then at 120 V for approximately 60–90 min through the resolving gel. Proteins were transferred onto PVDF membranes using wet transfer at 250 mA for 90 min at 4°C. After transfer, membranes were blocked with 5% skim milk in TBST for 1.5 h at room temperature.

Membranes were incubated with the following primary antibodies: anti-CB1R (Abclonal, Cat. No. A1447, 1:1000), anti-SREBP1 (Abclonal, Cat. No. A15586, 1:1000), anti-NAMPT (Abclonal, Cat. No. A0256, 1:1000), anti-SIRT1 (Proteintech, Cat. No. 13161-1-AP, 1:1000), and anti-GAPDH (Proteintech, Cat. No. 66009-1-Ig, 1:5000). Primary antibody incubation was performed overnight at 4°C. After washing with TBST, membranes were incubated with HRP-conjugated secondary antibody (Beyotime, Shanghai, China, Cat. No. A0208, 1:5000) for 1 h at room temperature.

Protein bands were visualized using an enhanced chemiluminescence detection reagent. Band intensities were quantified using ImageJ software, and target protein expression levels were normalized to GAPDH. Uncropped original Western blot images are provided in the Supplementary files.

**RT-qPCR**

Ipsilateral spinal dorsal horn tissues were collected for the detection of mRNA expression levels of CB1R, NAMPT, SREBP1, SIRT1, and β-actin. Briefly, dorsal horn tissue was minced and homogenized in 1 ml TRIzol reagent (Invitrogen, Carlsbad, CA, USA) using a homogenization tube containing four homogenization beads. Total RNA was extracted according to the manufacturer’s protocol. RNA concentration and purity were determined by UV spectrophotometry, and RNA integrity was assessed by agarose gel electrophoresis.

For reverse transcription, 1.0 μg of total RNA was first denatured at 65°C for 5 min and rapidly cooled on ice. cDNA was synthesized using M-MLV reverse transcriptase in a final reaction volume of 20 μl. The reverse transcription reaction was performed at 42°C for 60 min, followed by enzyme inactivation at 85°C for 5 min.

Quantitative real-time PCR was performed using a SYBR Green-based system on a CFX96 instrument (Bio-Rad, USA). Each 20 μl qPCR reaction contained 10 μl of 2× SYBR Green qPCR Master Mix, 0.4 μl of forward primer, 0.4 μl of reverse primer, 1 μl of cDNA template, and 8.2 μl of nuclease-free water. All reactions were performed in triplicate. The qPCR cycling conditions were as follows: initial denaturation at 95°C for 30 s, followed by 40 cycles of denaturation at 95°C for 15 s, annealing at 58°C for 20 s, and extension at 72°C for 30 s. A melting curve analysis was performed at the end of amplification to confirm product specificity.

Relative mRNA expression levels were calculated using the 2−ΔΔCt method, with β-actin used as the internal reference gene. Primers used for RT-qPCR were listed in the 5′→3′ direction as follows: for CB1R, forward 5′-AAGTCGATCTTAGACGGCCTT-3′ and reverse 5′-TCCTAATTTGGATGCCATGTCTC-3′; for NAMPT, forward 5′-TTTTCACGGCATTCAAAGTAGGA-3′ and reverse 5′-GCAGAAGCCGAGTTCAACATC-3′; for SREBP1, forward 5′-TGACCCGGCTATTCCGTGA-3′ and reverse 5′-CTGGGCTGAGCAATACAGTTC-3′; and for SIRT1, forward 5′-GCTGACGACTTCGACGACG-3′ and reverse 5′-TCGGTCAACAGGAGGTTGTCT-3′.

**Statistical analysis**

Sample sizes were calculated following a suggested study protocol. Statistical analysis was conducted using SPSS version 23.0 for variance analysis on Paw Withdrawal Threshold (PWT), Thermal Pain Threshold (TPT) tests, optical density (OD values) of immunoreactive neurons in the spinal dorsal horn (3 fields of view per slide at 200× magnification), and qPCR analysis of the spinal dorsal horn. Band absorbance values were analyzed, with the grayscale ratio of GAPDH serving as the relative expression level for target proteins. Data with normal distribution across groups indicated by the normality test are presented as mean ± standard deviation (SD). Variance analysis within and between groups treated with AM251 and Win55212-2 was performed. Longitudinal behavioral data (PWT and TPT) were analyzed using two-way repeated-measures ANOVA, with Time and Treatment as the two main factors, followed by Tukey’s post hoc test for multiple comparisons. A value of P < 0.05 was considered statistically significant. P < 0.05 was considered statistically significant.
