# Supplementary material for: Cannabinoid CB1 Receptor Regulates Spinal Dorsal Horn Synaptic Plasticity in a Mouse Model of Neuropathic Pain through the CB1R/SREBP1/NAMPT/SIRT1 Metabolic Regulatory Framework
Source: Brain Behav. 2026 Oct 13;16(10):e71837. doi: 10.1002/brb3.71837 (PMC13659934; doi:10.1002/brb3.71837)
Supplement: Supplementary file 2 [file BRB3-16-e71837-s002.docx]

**Table S1.** Changes in PWT from day 3 to day 14 across groups

|  | CCI+A group | shamCCI+A group | normal+A group | CCI+W group | sham CCI+W group | normal+W group |
| --- | --- | --- | --- | --- | --- | --- |
| Before treatment | 2.86±0.75 | 2.85±0.66 | 2.74±0.72 | 2.85±0.85 | 2.7±0.75 | 2.75±0.85 |
| After  3 days | 2.12±0.77 | 2.58±0.82 | 2.62±0.74 | 2.18±0.42 | 2.62±0.64 | 2.68±0.75 |
| After  7 days | 1.35±0.28^★※^ | 2.6±0.79 | 2.51±0.58 | 1.41±0.38^★※^ | 2.59±0.71 | 2.62±0.6 |
| After  14 days | 0.89±0.21^★※☆^ | 2.52±0.72 | 2.6±0.65 | 2.01±0.45 | 2.6±0.42 | 2.65±0.5 |

Data are presented as mean ± SD. n = 8 for CCI + AM251, n = 7 for sham CCI + AM251, n = 8 for normal + AM251, n = 9 for CCI + WIN55,212-2, n = 9 for sham CCI + WIN55,212-2, and n = 7 for normal + WIN55,212-2. PWT data were analyzed using two-way repeated-measures ANOVA followed by Tukey–Kramer post hoc test. The full analysis showed significant effects of group [F(8,59) = 9.74, P < 0.001], time [F(3,177) = 24.51, P < 0.001], and group × time interaction [F(24,177) = 8.59, P < 0.001]. ★P < 0.05 vs the corresponding sham CCI group; ※P < 0.05 vs the corresponding normal control group; ☆P < 0.05 vs the CCI + WIN55,212-2 group. CCI, chronic constriction injury; PWT, paw withdrawal threshold.

**Table S2**. Changes in Thermal Pain Threshold (TPT, paw withdrawal latency) from days 3 to 14 across all groups (s)

|  | CCI+A | shamCCI+A | normal+A | CCI+W | sham CCI+W | normal+W |
| --- | --- | --- | --- | --- | --- | --- |
| Before treatment | 12.7±4.8 | 12.6±5.2 | 12.5±4.3 | 12.6±4.9 | 12.7±5.3 | 12.4±4.8 |
|  |  |  |  |  |  |  |
| After 3 days | 9.5±3.4 | 12.5±4.1 | 12.3±4.4 | 9.7±3.2 | 12.3±4.3 | 12.4±4.4 |
| After 7days | 7.05±2.3^★※^ | 12.5±4.8 | 12.2±3.3 | 7.3±2.2^★※^ | 11.8±4.4 | 12.0±4.2 |
| After 14days | 4.25±1.0^★※☆^ | 12.2±4.3 | 11.9±3.4 | 9.1±1.6 | 11.85±4.6 | 12.0±4.1 |

Data are presented as mean ± SD. n = 8 for CCI + AM251, n = 7 for sham CCI + AM251, n = 8 for normal + AM251, n = 9 for CCI + WIN55,212-2, n = 9 for sham CCI + WIN55,212-2, and n = 7 for normal + WIN55,212-2. Thermal withdrawal latency data were analyzed using two-way repeated-measures ANOVA followed by Tukey–Kramer post hoc test. The full analysis showed significant effects of group [F(8,59) = 9.28, P < 0.001], time [F(3,177) = 22.51, P < 0.001], and group × time interaction [F(24,177) = 9.59, P < 0.001]. ★P < 0.05 vs the corresponding sham CCI group; ※P < 0.05 vs the corresponding normal control group; ☆P < 0.05 vs the CCI + WIN55,212-2 group. CCI, chronic constriction injury.

**Table S3.** LTP amplitude changes in A-fiber–evoked responses in the spinal dorsal horn measured at 60 minutes after HFS induction on day 14.

|  | CCI+A group | ShamCCI+A group | normal+A group | CCI+W group | sham CCI+W group | normal+W group |
| --- | --- | --- | --- | --- | --- | --- |
| Before stimulation | 17.4±3.8 | 12.4±2.9 | 14.1±3.6 | 13.7±3.8 | 14.0±3.8 | 13.9±3.1 |
| After stimulation | 59.5±12.3^★※☆^ | 12.7±5.6 | 15.8±4.9 | 14.5±3.2 | 15.3±5.8 | 14.8±7.3 |
| Percentage difference before and after stimulation | 240.9.3% | 2.4% | 12.6% | 5.8% | 9.3% | 6.5% |
|  | t<0.01 | t>0.05 | t>0.05 | t>0.05 | t>0.05 | t>0.05 |

Data are presented as mean ± SD. n = 8 for CCI + AM251, n = 7 for sham CCI + AM251, n = 8 for normal + AM251, n = 9 for CCI + WIN55,212-2, n = 9 for sham CCI + WIN55,212-2, and n = 7 for normal + WIN55,212-2. Post-HFS values correspond to responses measured at 60 min after HFS induction. Within-group comparisons between baseline and post-HFS values were analyzed using paired t-tests. Between-group comparisons of post-HFS values were analyzed using one-way ANOVA followed by Tukey–Kramer post hoc test. Percentage difference was calculated as [(post-HFS value − baseline value) / baseline value] × 100%. HFS, high-frequency stimulation. ★P < 0.05 or P < 0.01 vs the corresponding sham and normal control groups; ※P < 0.05 or P < 0.01 vs the corresponding pre-HFS baseline within the same group; ☆P < 0.01 vs the CCI + WIN55,212-2 group.

**Table S4**. LTP response area changes in A-fiber–evoked responses in the spinal dorsal horn measured at 60 minutes after HFS induction on day 14.

|  | CCI+A group | shamCCI+A group | normal+A group | CCI+W group | sham CCI+W group | normal+W group |
| --- | --- | --- | --- | --- | --- | --- |
| Before stimulation | 47.8±8.5 | 43.4±9.4 | 43.1±7.8 | 42.4±8.7 | 44.5±9.7 | 43.6±9.4 |
| After stimulation | 91.6±18.7^★※☆^ | 45.6±9.9 | 45.3±8.9 | 43.2±9.4 | 45.2±9.5 | 45.8±8.2 |
| Percentage difference before and after stimulation  （%） | 92.6%  t<0.01 | 5.7%  t>0.05 | 5.1%  t>0.05 | 1.9%  t>0.05 | 1.2%  t>0.05 | 5.1%  t>0.05 |

Data are presented as mean ± SD. n = 8 for CCI + AM251, n = 7 for sham CCI + AM251, n = 8 for normal + AM251, n = 9 for CCI + WIN55,212-2, n = 9 for sham CCI + WIN55,212-2, and n = 7 for normal + WIN55,212-2. Post-HFS values correspond to responses measured at 60 min after HFS induction. Within-group comparisons between baseline and post-HFS values were analyzed using paired t-tests. Between-group comparisons of post-HFS values were analyzed using one-way ANOVA followed by Tukey–Kramer post hoc test. Percentage difference was calculated as [(post-HFS value − baseline value) / baseline value] × 100%. HFS, high-frequency stimulation. ★P < 0.05 or P < 0.01 vs the corresponding sham and normal control groups; ※P < 0.05 or P < 0.01 vs the corresponding pre-HFS baseline within the same group; ☆P < 0.01 vs the CCI + WIN55,212-2 group.

**Table S5.** LTP amplitude changes in C-fiber–evoked responses in the spinal dorsal horn measured at 60 minutes after HFS induction on day 14.

|  | CCI+A group | ShamCCI+A group | normal+A group | CCI+W group | sham CCI+W group | normal+W group |
| --- | --- | --- | --- | --- | --- | --- |
| Before stimulation | 14.2±3.8 | 12.7±3.2 | 11.8±4.6 | 13.7±3.8 | 14.0±3.8 | 13.9±3.1 |
| After stimulation | 42.4±7.8^#★※☆^ | 27.7±5.5^#★※^ | 22.8±4.9^#★※^ | 21.5±4.2^#★※^ | 25.3±5.8^#★※^ | 24.8±7.3^#★※^ |
| Percentage difference before and after stimulation（%） | 198.6%  t<0.001 | 118%  t<0.01 | 93.2%  t<0.01 | 57.1%  t<0.05 | 84.8%  t<0.01 | 78.4%  t<0.01 |

Data are presented as mean ± SD. n = 8 for CCI + AM251, n = 7 for sham CCI + AM251, n = 8 for normal + AM251, n = 9 for CCI + WIN55,212-2, n = 9 for sham CCI + WIN55,212-2, and n = 7 for normal + WIN55,212-2. Post-HFS values correspond to responses measured at 60 min after HFS induction. Within-group comparisons between baseline and post-HFS values were analyzed using paired t-tests. Between-group comparisons of post-HFS values were analyzed using one-way ANOVA followed by Tukey–Kramer post hoc test. Percentage difference was calculated as [(post-HFS value − baseline value) / baseline value] × 100%. HFS, high-frequency stimulation. ★P<0.01 for the CCI+A group versus sham+A and normal+A groups. #P<0.05 for the CCI+W group versus sham+W and normal+W groups. ※P<0.05 or 0.01 when compared to before rigid electrical stimulation within the same group. ☆P<0.01 for the CCI+A group versus the CCI+W group.

**Table S6.** LTP response area changes in C-fiber–evoked responses in the spinal dorsal horn measured at 60 minutes after HFS induction on day 14.

|  | CCI+A group | shamCCI+A group | normal+A group | CCI+W group | sham CCI+W group | normal+W group |
| --- | --- | --- | --- | --- | --- | --- |
| Before stimulation | 59.5±15.2 | 56.2±12.7 | 56.9±14.6 | 55.4±8.7 | 55.5±9.7 | 56.6±9.4 |
| After stimulation | 105.7±26.1^★#※☆^ | 68.5±15.8^★#※^ | 62.4±16.2^★#※^ | 60.5±9.9^★#※^ | 66.2±9.5^★#※^ | 64.2±8.2^★#※^ |
| Percentage difference before and after stimulation（%） | 77．6% | 17.9%  t<0.05 | 10.9%  t<0.05 | 5.1%  t>0.05 | 16.5%  t<0.05 | 13.5%  t<0.05 |

Data are presented as mean ± SD. n = 8 for CCI + AM251, n = 7 for sham CCI + AM251, n = 8 for normal + AM251, n = 9 for CCI + WIN55,212-2, n = 9 for sham CCI + WIN55,212-2, and n = 7 for normal + WIN55,212-2. Post-HFS values correspond to responses measured at 60 min after HFS induction. Within-group comparisons between baseline and post-HFS values were analyzed using paired t-tests. Between-group comparisons of post-HFS values were analyzed using one-way ANOVA followed by Tukey–Kramer post hoc test. Percentage difference was calculated as [(post-HFS value − baseline value) / baseline value] × 100%. HFS, high-frequency stimulation. ★P<0.01 for the CCI+A group versus sham+A and normal+A groups. #P<0.05 for the CCI+W group versus sham+W and normal+W groups. ※P<0.05 or 0.01 when compared to before rigid electrical stimulation within the same group. ☆P<0.01 for the CCI+A group versus the CCI+W group.

**Table S7.** Quantitative results of TG, TC, LDL, and HDL profiles (mmol/L) in plasma in different groups

|  | CCI+A | shamCCI+ A | normal+ A | CCI+W | sham CCI+ W | normal+ W |
| --- | --- | --- | --- | --- | --- | --- |
| TG | 1.1±0.5^★☆^ | 1.4±0.5 | 1.2±0.6 | 2.85±0.5^※^ | 1.5±0.7 | 1.4±0.7 |
| TC | 2.78±0.3^★☆^ | 4.6±0.4 | 4.3±0.3 | 6.8±0.5^※^ | 5.0±0.3 | 4.7±0.4 |
| LDL | 1.4±0.5^★☆^ | 2.4±0.5 | 2.2±0.35 | 3.10±0.6^※^ | 2.5±0.45 | 2.6±0.4 |
| HDL | 1.9±0.7^★☆^ | 0.8±0.5 | 0.95±0.6 | 0.6±0.2^※^ | 0.9±0.3 | 0.85±0.25 |

Data are presented as mean ± SD. n = 8 for CCI + AM251, n = 7 for sham CCI + AM251, n = 8 for normal + AM251, n = 9 for CCI + WIN55,212-2, n = 9 for sham CCI + WIN55,212-2, and n = 7 for normal + WIN55,212-2. Plasma lipid indices were compared among groups using one-way ANOVA followed by Tukey–Kramer post hoc test. ★P < 0.05 or P < 0.01 vs the sham CCI + AM251 and normal + AM251 groups; ※P < 0.05 or P < 0.01 vs the sham CCI + WIN55,212-2 and normal + WIN55,212-2 groups; ☆P < 0.01 vs the CCI + WIN55,212-2 group. TG, triglycerides; TC, total cholesterol; LDL, low-density lipoprotein cholesterol; HDL, high-density lipoprotein cholesterol.

**Table S8.** Mean optical density (MOD) values of CB1R, NAMPT, SREBP1, and SIRT1 immunohistochemical staining in the ipsilateral spinal dorsal horn

|  | CCI+A | shamCCI+A | normal+A | CCI+W | sham CCI+W | normal+W |
| --- | --- | --- | --- | --- | --- | --- |
| CB1R | 32.6±8.05^★☆^ | 40.4±9.5 | 43.8±11.4 | 69.5±9.2^※^ | 49.2±10.6 | 45.3±18.6 |
| SREBP1 | 42.7±9.2^★☆^ | 62.2±9.8 | 68.3±10.5 | 80.7±12.6^※^ | 59.3±15.2 | 54.2±12.7 |
| NAMPT | 34.7±15.5^★☆^ | 52.3±15.1 | 59.6±20.1 | 65.6±18.3^※^ | 57.3±14.7 | 53.6±18.8 |
| SIRT1 | 88.2±7.4^★☆^ | 74.5±5.9 | 78.6±6.3 | 39.6±5.8^※^ | 69.2±11.6 | 62.5±9.7 |

Data are presented as mean ± SD. n = 8 for CCI + AM251, n = 7 for sham CCI + AM251, n = 8 for normal + AM251, n = 9 for CCI + WIN55,212-2, n = 9 for sham CCI + WIN55,212-2, and n = 7 for normal + WIN55,212-2. Plasma lipid indices were compared among groups using one-way ANOVA followed by Tukey–Kramer post hoc test. ★P < 0.05 or P < 0.01 vs the sham CCI + AM251 and normal + AM251 groups; ※P < 0.05 or P < 0.01 vs the sham CCI + WIN55,212-2 and normal + WIN55,212-2 groups; ☆P < 0.01 vs the CCI + WIN55,212-2 group. TG, triglycerides; TC, total cholesterol; LDL, low-density lipoprotein cholesterol; HDL, high-density lipoprotein cholesterol.
